# Dispersive nonreciprocity between a qubit and a cavity

**DOI:** 10.1126/sciadv.adj8796

**Published:** 2024-04-17

**Authors:** Ying-Ying Wang, Yu-Xin Wang, Sean van Geldern, Thomas Connolly, Aashish A. Clerk, Chen Wang

**Affiliations:** ^1^ Department of Physics, University of Massachusetts-Amherst, Amherst, MA, USA.; ^2^ Pritzker School of Molecular Engineering, University of Chicago, Chicago, IL, USA.

## Abstract

The dispersive interaction between a qubit and a cavity is ubiquitous in circuit and cavity quantum electrodynamics. It describes the frequency shift of one quantum mode in response to excitations in the other and, in closed systems, is necessarily bidirectional, i.e., reciprocal. Here, we present an experimental study of a nonreciprocal dispersive-type interaction between a transmon qubit and a superconducting cavity, arising from a common coupling to dissipative intermediary modes with broken time reversal symmetry. We characterize the qubit-cavity dynamics, including asymmetric frequency pulls and photon shot noise dephasing, under varying degrees of nonreciprocity by tuning the magnetic field bias of a ferrite component in situ. We introduce a general master equation model for nonreciprocal interactions in the dispersive regime, providing a compact description of the observed qubit-cavity dynamics agnostic to the intermediary system. Our result provides an example of quantum nonreciprocal phenomena beyond the typical paradigms of non-Hermitian Hamiltonians and cascaded systems.

## Teaser

In a generalization of the dispersive interaction, a qubit and a cavity interact directionally without excitation exchange.

## INTRODUCTION

Theoretical and experimental studies of nonreciprocity are of great interest, both due to their fundamental implications for realizing exotic phases of matter (*1*–*3*) and their relevance to applications in classical and quantum information processing. The most widely investigated nonreciprocal phenomena concerns the scattering matrix *S* of input and output signals for a multiport network, where the transmission coefficient is not invariant under the exchange of the source and the receiver, i.e., *S_ij_* ≠ *S_ji_*, with isolators and circulators being the canonical examples. Realization of nonreciprocal scattering in optical (*4*, *5*), acoustic (*6*, *7*), and microwave domains (*8*, *9*) with new techniques has been an intensive area of study. On the other hand, the concept of nonreciprocity goes beyond the scattering properties of propagating linear modes, especially in quantum contexts where one often considers nonreciprocal interactions between stationary quantum subsystems. When the stationary modes are linear and in the classical correspondence limit, their nonreciprocal interactions can be conveniently described by a non-Hermitian Hamiltonian (*5*, *6*, *10*, *11*). Such non-Hermitian dynamics have been shown in recent works to generate a plethora of interesting physical phenomena, including the non-Hermitian skin effect (*10*–*12*) and previously unexplored critical phenomena under monitored dynamics (*13*, *14*).

Nonreciprocal interactions that are more uniquely quantum arise when the subsystems of interest include strongly nonlinear modes, which necessitates the use of master equations to describe the system dynamics. A well-studied example is a cascaded network of resonant qubits (*15*), where the interaction between neighboring qubits is mediated by emission and absorption via a directional waveguide (*16*). The resulting effective interaction can be described as nonreciprocal transfer of single excitations. This cascaded model has been investigated theoretically, where the nonreciprocity leads to unique dynamics such as steady-state entanglement and dimerized many-body states. Experimental development in chiral quantum optical platforms (*17*–*19*) and waveguide circuit quantum electrodynamics (QED) (*20*–*23*) are expected to realize such resonant nonreciprocal phenomena in the near future.

It is natural to consider nonreciprocal interactions that go beyond (one-way) excitation transfer, as is expected if the relevant subsystems are nonresonant. This is particularly relevant in quantum device engineering where it is commonplace to use weak hybridization of disparate linear and nonlinear modes. For example, the dispersive Hamiltonian between a qubit and a cavity, $\frac{\hbar \chi }{2}{\hat{a}}^{†}\hat{a}{\hat{\sigma }}_{z}$ , forms the cornerstone of circuit QED and superconducting quantum computation (*24*). Here, the qubit or the cavity experiences a frequency shift in response to an excitation in one other, by the same amount χ, exemplifying the reciprocal nature of the dispersive interaction in a closed quantum system. While the dispersive Hamiltonian is an approximate effective model of the underlying Jaynes-Cummings type couplings, its ability to compactly describe prominent experimental observables without needing all microscopic details makes it an extremely valuable tool in describing light-matter interactions.

Recently, a class of nonreciprocal interactions that is distinct from the cascaded quantum systems has been theoretically investigated (*25*). The simplest example, arising from dispersive-type couplings in open quantum systems, can be described by a single Lindblad dissipator, $D\left[\hat{a}e^{i\theta {\hat{\sigma }}_{z}}\right]$ , which leads to a one-way influence of a cavity mode $\hat{a}$ on a qubit ${\hat{\sigma }}_{z}$ in terms of a phase or frequency shift. This leads to several basic questions. What is the generic effective model of open-system qubit-cavity interactions in the dispersive regime? How robustly can we model the nonreciprocal features of such interactions in practical systems in the presence of complex microscopic details and imperfections?

In this work, we experimentally realize and characterize a dispersive type of quantum nonreciprocal interaction between a superconducting cavity and a transmon qubit. To access varying degrees of nonreciprocity in situ, we implement a hybrid quantum system where the qubit-cavity interaction is mediated by a complex set of cavity-magnon modes constructed from three-dimensional niobium and copper waveguide cavities and a ferrimagnetic yttrium iron garnet (YIG) crystal (*26*–*29*). We study the nonreciprocal influence between the qubit and the cavity in terms of asymmetric dispersive frequency shifts and impacts on dephasing and decay rates. Our experiment provides an example of generalized dispersive interaction in open quantum systems.

A central conclusion of our study is that the nonreciprocal features of dispersive-type interactions in an open system can be encapsulated by a simple Lindblad dissipator

$$D\;\left[e^{\frac{i\theta +\eta }{2}{\hat{\sigma }}_{z}}\hat{a}\right]$$ (1)

This nontrivial extension of (*25*) applies to an extremely wide class of microscopic dissipative bath models (i.e., a general linear network of lossy modes) and allows a few-parameter description of the effective qubit-cavity interaction with full predictive power of the resulting quantum dynamics. We have validated this in our experiments.

While optimal methods for engineering desirable interaction properties (specified by θ and η in Eq. 1) remain to be explored in future work, our study establishes a practical characterization and analysis framework for describing the phenomena of dispersive nonreciprocity.

## RESULTS

In this main section, we will first introduce the experimental system setup and present observations of dispersive qubit-cavity frequency shifts with varying degrees of nonreciprocity through Ramsey measurements. Then, we present a general master equation model of dispersive nonreciprocity with dissipator Eq. 1 and experimentally characterize the model parameters from measured free-evolution dynamics of the system starting from an initial cavity coherent state. Last, we validate and demonstrate the utility of the model in predicting system dynamics under different control protocols such as continuous cavity drive and Fock state generation.

### System setup

**Fig. 1.**
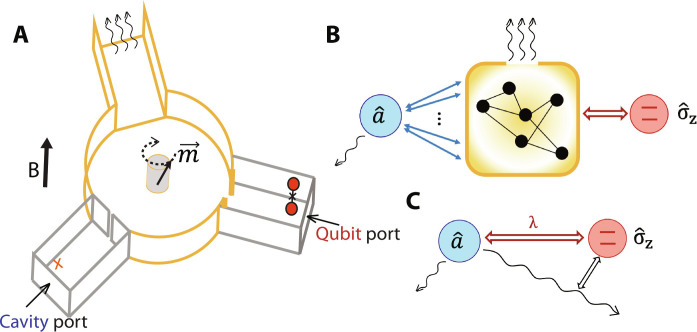
Schematics of our integrated nonreciprocal device. (**A**) A cartoon drawing of our device (not to scale), composed of a Cu waveguide Y-junction loaded with a YIG cylinder, two close-ended Nb rectangular waveguide segments with weakly coupled drive ports, named as the “cavity port” and the “qubit port,” respectively, in the figure. The bottom left segment houses the cavity mode under study and an ancilla transmon (small orange cross) to facilitate readout of the cavity state. The right segment houses the transmon qubit under study. The magnetic dipole in the YIG crystal is coupled to a series of microwave modes through its precession under external magnetic field. The top waveguide segment of the Y-junction is impedance-matched to a transmission line for signal output. (**B**) A general schematic of the mode connectivity of system, where the cavity is exchange-coupled to many intermediary modes and the qubit is dispersively coupled to them. The intermediary modes contain a large collective loss inherited from the open waveguide port. (**C**) Schematic representation of the qubit-cavity effective model, Eq. 2, with their reciprocal dispersive coupling λ and a dispersive type of nonreciprocal dissipation operator $\Gamma D\left[e^{\frac{i\theta +\eta }{2}{\hat{\sigma }}_{z}}\hat{a}\right]$.

Our experimental setup is shown in Fig. 1A. By inserting a single-crystalline YIG cylinder into the center of a copper waveguide Y-junction and applying a variable external magnetic field, we engineer a nonreciprocal interaction between a niobium superconducting cavity and a superconducting transmon qubit connected to two output ports of the waveguide Y-junction (see Materials and Methods for details). The level of directionality can be tuned in situ via the external field. At an external bias of about ±20 mT, the ferrite-loaded Y-junction functions like a circulator that mediates directional microwave transmission between the cavity side and the transmon side with a bandwidth of a few hundred megahertz (*26*).

Unlike in a practical circulator where broadband directional isolation is usually a first priority, the most important quality of our device is the low internal loss of the cavity and magnon modes. Therefore, we can describe the entire intermediary system (except for the cavity and the qubit themselves) as a network of coupled linear modes that share only one dominant decay channel, the 50-ohm transmission line at the third port of the waveguide Y-junction, as shown in Fig. 1 (A and B), regardless of the external field. This allows us to treat the linear network, whose underlying details prove too challenging to characterize precisely, as a single dissipative bath in mediating the effective interaction between the longer-lived qubit and cavity. It is such a generalized quantum interaction, which encompasses both the standard dispersive interaction (*24*) and the dissipative directional effects as represented by Fig. 1C, that is the subject of our study.

Integration of superconducting qubits in a hybrid quantum system with ferromagnetic magnons faces substantial challenges in mitigating the impact of magnetic fields on qubit coherence. Previous experiments used permanent magnets to provide strong local bias fields away from the qubit, achieving qubit lifetimes ranging from <1 μs (*30*–*32*) to about 3 μs recently (*33*) but have limited to no tunability of external field. In our experiment, we use the large electromagnet of the cryostat to apply a global magnetic field to the device, sufficient to fully reverse the directionality of the qubit-cavity coupling. At the same time, the qubit is shielded by the Meissener effect of a niobium waveguide and a layer of high-permeability foil. We observe *T*_1_ and *T*_2_ on the order of a few microseconds (up to 10 μs) while being unaffected by the applied field up to ±0.1 T. While future implementation of tunable nonreciprocity in circuit QED may ultimately benefit from alternative strategies based on Josephson parametric circulators (*9*, *34*, *35*), our ferrite-based platform avoids the outstanding challenges of parametric pump tone leakage that could severely degrade qubit coherence and obscure the dispersive effects with spurious Stark shifts.

The cavity in our experiment is evanescently coupled to the Y-junction through an aperture, whose loss is dominated by coupling to the Y-junction. The cavity can be driven from a weakly coupled cavity port as labeled in Fig. 1A. An additional transmon (marked as an orange ×) is installed inside the cavity for readout of the cavity state. This additional transmon will not participate in the quantum dynamics under study and will be referred to as the ancilla, to distinguish it from the qubit. On the other hand, the qubit can be driven from a weakly coupled qubit port as labeled in Fig. 1A. The qubit is Purcell protected by effectively a buffer cavity mode formed by a modest constriction slot between the niobium waveguide segment and the Y-junction. This extra buffer mode is sufficiently short-lived to be treated as part of the dissipative bath rather than a quantum object, but it provides an impedance environment to boost the interaction between the qubit and the cavity.

### Observation and control of nonreciprocal frequency shifts

As the qubit and cavity are detuned, we expect the mediated interaction to be dispersive. To characterize the phenomenon of dispersive nonreciprocity between the qubit and the cavity, we compare the qubit frequency shift per cavity photon, labeled χ_cq_, with cavity frequency shift in response to the qubit excitation, labeled χ_qc_. A closed quantum system is reciprocal, where the dispersive frequency pull per excitation in both directions are guaranteed to be equal, i.e., χ_cq_ = χ_qc_. Violation of this relation is a sign of nonreciprocity.

**Fig. 2.**
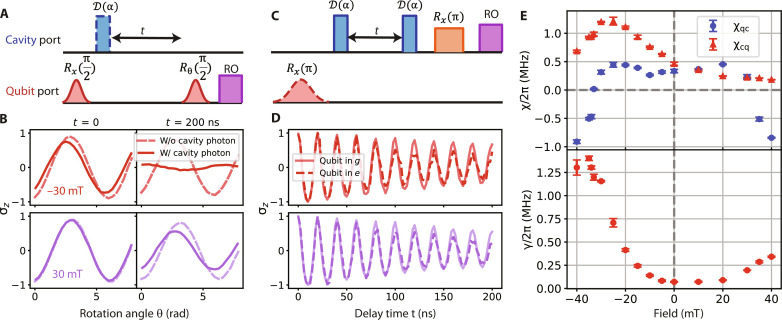
Demonstration of nonreciprocal qubit-cavity frequency shifts. (**A**) The pulse diagram and (**B**) example results of a qubit Ramsey measurement under coherent cavity photon population at selective magnetic field (*B* = ±30 mT). The qubit coherence evolution is obtained by initializing the qubit in an equator state and cavity in a coherent state with a displacement pulse *D*(α). The system then evolves over a wait time *t* followed by a second π/2 qubit rotation along a variable rotation angle θ and a readout (RO) of the qubit state. The qubit state against rotation angle θ yields a sinusoidal graph as plotted in (B), where the phase and amplitude can be extracted. The solid and dashed lines show the qubit Ramsey curves with and without cavity photons, respectively. (**C**) The pulse diagram and (**D**) the experimental result of the cavity photon Ramsey measurement with a relatively long ancilla excitation pulse [orange *R_x_*(π)] to obtain the qubit-dependent cavity frequency, where the solid (dashed) lines correspond to the qubit in |*g*〉 (|*e*〉). (**E**) The top plots both measured χs under different external magnetic fields showing variable nonreciprocity between the cavity and the qubit. χ_qc_ is near-symmetric across the external field, as apparent in (D), where the −30- and 30-mT curves are similar to each other. χ_cq_ is clearly asymmetric, where the positive and negative field results are distinctly different, as shown in (B). The bottom is the qubit dephasing rate under different external magnetic fields.

To measure χ_cq_, we perform Ramsey experiments on the qubit with and without cavity photons. By comparing these measurements, we can extract the accumulated extra qubit phase shift ϕ caused by cavity photons over a finite time window of *t* = 200 ns, as shown in Fig. 2A. The cavity is initialized in a coherent state with an initial mean photon number of ${\bar{n}}_{0}\approx 3$ . The choice of ${\bar{n}}_{0}$ and *t* is motivated by optimizing signal-to-noise ratio while keeping the cavity well within the linear regime. The cavity photon number undergoes free decay over time, and hence, the instantaneous qubit frequency also varies over time. Assuming the dispersive frequency shift is proportional to photon numbers (as is the case in closed-system circuit QED), we define the cavity-to-qubit dispersive shift $\chi_{\text{cq}}=\phi /\left(t\cdot {\bar{n}}_{\text{avg}}\right)$ , where ${\bar{n}}_{\text{avg}}$ is the time-averaged cavity photon number during the integration time *t*. Example results of the qubit state versus rotation angle θ of the second Ramsey π/2 pulse are shown in Fig. 2B (solid lines), which informs the amplitude and phase of the qubit coherence function 〈σ_−_(*t*)〉 at fixed time *t*. By comparing 〈σ_−_(*t*)〉 to the reference qubit state $\langle \sigma_{-}^{0}\left(t\right)\rangle$ measured without cavity photons (dashed lines) at the same *t*, we can extract the photon-induced phase shift ϕ and decoherence factor ζ at time *t* from

$\left\langle \sigma_{-}\right\rangle /\left\langle \sigma_{-}^{0}\right\rangle =\zeta e^{i\phi }$ . The time-averaged cavity photon number ${\bar{n}}_{\text{avg}}$ is calibrated using a separate Ramsey experiment of the ancilla inside the cavity for the same time window, which allows for normalization of χ_cq_ as a per-photon quantity (see Materials and Methods for details of the calibration). The extracted decoherence factor ζ informs the cumulative loss of qubit coherence due to photon shot noise over the time window *t*, which can be similarly converted to a qubit dephasing rate per photon, $\gamma =-\text{ln}\left(\zeta \right)/\left(t\cdot {\bar{n}}_{\text{avg}}\right)$.

On the other hand, χ_qc_ is measured with a cavity Ramsey protocol with the qubit in its ground (|*g*〉 ≡ |↑〉) or excited (|*e*〉 ≡ |↓〉) state. The cavity Ramsey sequence is composed of two cavity displacement pulses with a wait time *t* in between, as shown in Fig. 2C. We use the dispersively coupled ancilla inside the cavity to read out the cavity state. This readout, inspired by photon number measurements in the strong dispersive regime (*36*, *37*), is implemented by a relatively long (spectrally narrow) ancilla π-pulse, followed by reading out the state of the ancilla. Example results of the ancilla state over *t*, representing the oscillation and decay of the cavity coherent state, are plotted in Fig. 2D, with the solid and dashed lines corresponding to the qubit in |*g*〉 and |*e*〉 states, respectively. Since the qubit lifetime *T*_1_ ≫ *t*, the qubit state does not change over the measurement window to a good approximation, as confirmed by the constant cavity oscillation frequency in this measurement. The extracted cavity frequency ωg and ωe gives χ_qc_ = ωg − ωe.

The two types of Ramsey measurements are carried out for various external magnetic fields, and the extracted dispersive shifts in both directions, χ_qc_ and χ_cq_, are plotted in the top of Fig. 2E. The magnetic field serves as a control knob to vary the complex bath-mediated qubit-cavity interaction. While it is not unexpected that nonreciprocity exists in the presence of magnetic field, our experiment presents an unambiguous signature of nonreciprocity in the dispersive regime, χ_qc_ ≠ χ_cq_, and demonstrates in situ control over the degree of such nonreciprocity, e.g., ranging from approximately reciprocal near-zero field to strongly nonreciprocal at high negative fields. Moreover, we note a few nontrivial features in the magnetic field dependence. First, χ_qc_ shows symmetry with respect to *B*; this is a result of an Onsager-type constraint on eigenvalues of a linear non-Hermitian Hamiltonians, which in turn arises from microscopic time-reversal symmetry even in the presence of external field. In contrast, χ_cq_ and the photon-induced dephasing rate γ (the bottom of Fig. 2E) show no such symmetry; this can be attributed to their sensitivity to the eigenvectors of the non-Hermitian Hamiltonian (see section S1C). Second, the nonreciprocity at zero field is small but definitely nonzero within experimental uncertainties. While many experiments have realized magnetless nonreciprocity by engineering synthetic flux (*35*, *38*), our data at zero field demonstrate an interesting theoretical aspect of quantum nonreciprocity: The dissipative interaction between quantum subsystems can be nonreciprocal without real or synthetic magnetic field (*25*).

### Effective nonreciprocal model in the dispersive regime

While the phenomenological observables χ_qc_ and χ_cq_ highlight a distinctive aspect of the effective qubit-cavity dispersive interaction, they do not a priori fully characterize the general dynamics of the qubit-cavity system. Given an arbitrary initial state of this qubit-cavity system, how can we model the system to fully predict its time evolution? Of course, if we know the full details of all relevant intermediary modes (Fig. 1B), including their mode frequencies, decay rates, internal and external coupling rates, then one could, in principle, solve the dynamics of the expanded system. However, not only is this approach computationally expensive and intuitively opaque, it is often unrealistic to extract detailed knowledge of a highly-dissipative multimode system. On the other hand, if the system is in a regime that allows (i) adiabatic elimination of the intermediary modes and (ii) dispersive approximation of the qubit, then we can derive a simple effective Markovian master equation only involving the cavity *a* and the qubit:

$$\begin{array}{c} \partial_{t}\hat{\rho }=-i[\Delta_{c}{\hat{a}}^{†}\hat{a}+\frac{\lambda }{2}{\hat{\sigma }}_{z}{\hat{a}}^{†}\hat{a},\hat{\rho }]+\\ \kappa D\left[\hat{a}\right]\hat{\rho }+\Gamma D\left[e^{\frac{i\theta +\eta }{2}{\hat{\sigma }}_{z}}\hat{a}\right]\hat{\rho } \end{array}$$ (2)

which is written in the rotating frame of both the qubit and a reference frequency of the cavity [see Materials and Methods and section S1 (A and B) for details] and can be fully specified via six independent real parameters. We have not included here the intrinsic decoherence of the qubit that would give rise to additional qubit-only dephasing and relaxation dissipators.

We now provide physical intuition for the emergence of Eq. 2 from the underlying microscopic model in Fig. 1B. As mode *a* is coupled to a linear network of bath modes, the effective cavity detuning Δc, cavity decay rate κ, and the conventional reciprocal dispersive interaction $\frac{\lambda }{2}{\hat{\sigma }}_{z}{\hat{a}}^{†}\hat{a}$ are expected. The nonlinear collective dissipator $D\;\left[e^{\frac{i\theta +\eta }{2}{\hat{\sigma }}_{z}}\hat{a}\right]$ , in contrast, describes a form of bath-mediated nonreciprocal interaction between the qubit and the cavity. It is instructive to consider this dissipator in two special limits

**Fig. 3.**
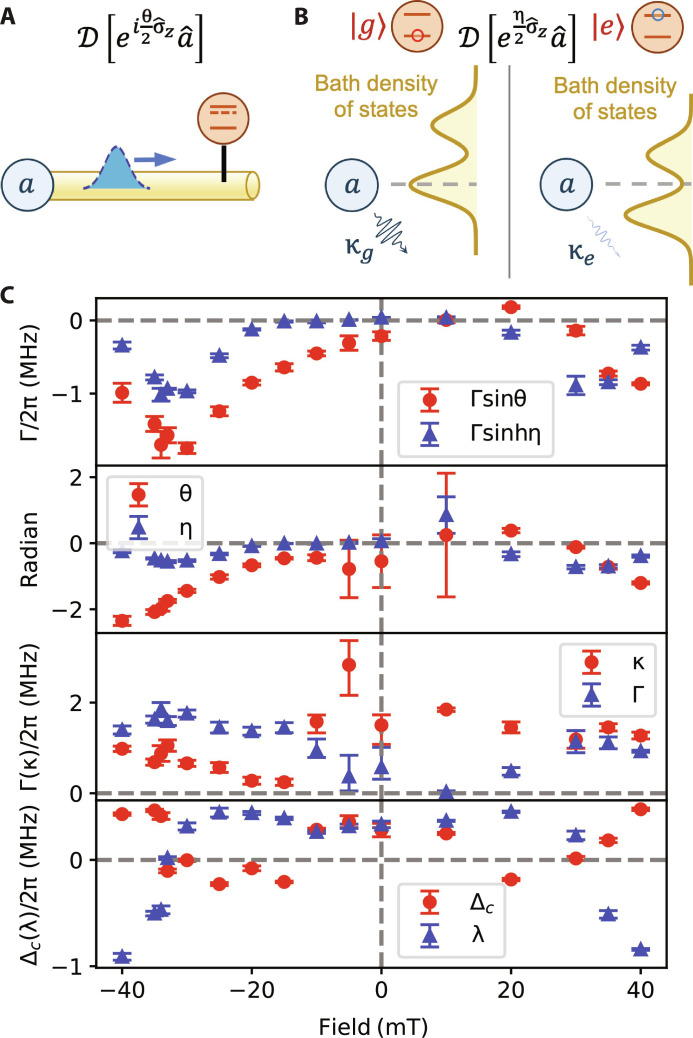
Schematic representation and experimental parameters of the master equation. (**A**) The case of θ ≠ 0, η = 0: The dissipator describes a fully directional dispersive interaction from the cavity to the qubit, which comes from the one-way traveling photon in the intermediary modes (the blue dashed wave packet) suppressing the qubit transition energy. (**B**) The case of θ = 0, η ≠ 0: The dissipator describes a qubit-state–dependent cavity decay process, which comes from density-of-states function of the intermediary mode being dispersively shifted by the qubit. (**C**) The six master equation parameters experimentally determined at different magnetic fields via Eqs. 12 to 17 (see Materials and Methods). We also show Γsinθ, which is the instantaneous one-way dispersive shift in the case of (A), and Γsinhη, which is the qubit-state dependence of cavity decay rate in the case of (B). Note that the parameter λ is equivalent to χ_qc_ in Fig. 2E.

**Fig. 4.**
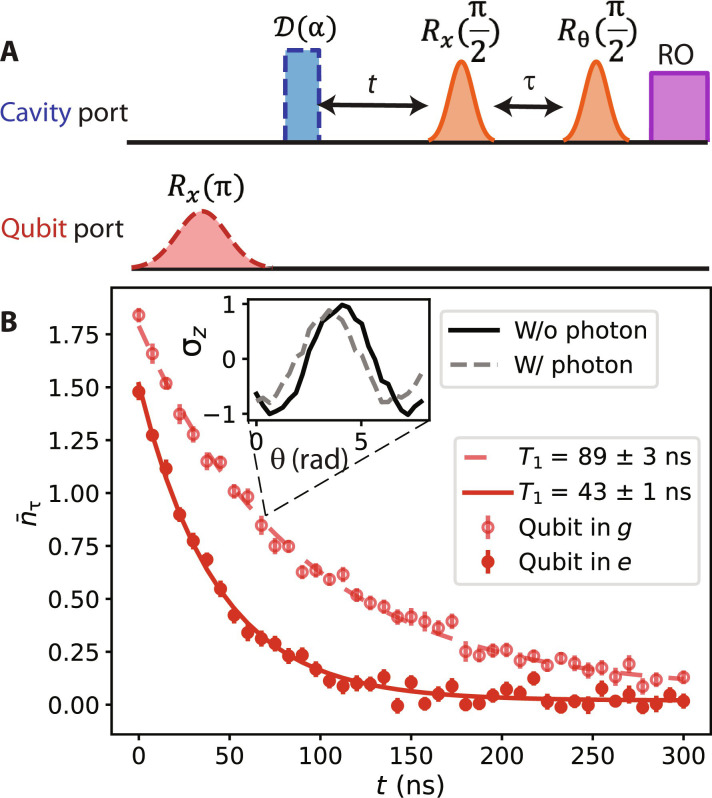
Measurements of qubit-state–dependent cavity decay rates. (**A**) The pulse diagram and (**B**) example result (*B* = −30 mT) of cavity decay rate measurement, with qubit prepared in |*g*〉 or |*e*〉. The cavity is prepared in a coherent state with a displacement pulse *D*(α). We map the cavity photon number to the ancilla’s phase shift ϕ over a sliding time window of fixed length (τ = 100 ns), which is measured in a Ramsey sequence of the ancilla. Although the instantaneous cavity photon number changes substantially during the time window τ, the cumulative phase shift can be used to infer the average photon number ${\bar{n}}_{\tau }$ during τ. ${\bar{n}}_{\tau }$ can be fit to an exponential decay as the Ramsey window slides in time *t* for both the qubit in |*g*〉 and in |*e*〉, which gives the cavity decay rates for the respective cases.

1) θ ≠ 0, η = 0: The dissipator $D\;\left[e^{\frac{i\theta }{2}{\hat{\sigma }}_{z}}\hat{a}\right]$ describes a fully directional dispersive interaction from the cavity to the qubit. The instantaneous one-way dispersive shift is Γsinθ per photon (c.f., Fig. 3C). At a heuristic level, this interaction is due to processes where cavity photons hop to the intermediary modes, briefly interact with the qubit, and then leak out to the environment (Fig. 3A). This dissipator has the general form $D\left[\hat{a}{\hat{U}}_{B}\right]$ (where ${\hat{U}}_{B}$ is a unitary operator on a subsystem *B*). As discussed in (*25*), general dissipators of this form exploit a dissipative gauge symmetry to realize one-way interactions.

2) θ = 0, η ≠ 0: The dissipator $D\left[e^{\frac{\eta }{2}{\hat{\sigma }}_{z}}\hat{a}\right]$ describes a qubit-state–dependent cavity decay process. For an example observation of this effect, see Fig. 4. We can view the multimode intermediary structure together with the qubit as a dissipative environment for the cavity (see Fig. 3B). As the qubit dispersively shifts the intermediary modes and hence the density-of-states function of the bath, the cavity decay rate (governed by the Fermi’s golden rule) acquires a qubit-state–dependent term of Γsinhη. As a backaction of this effect, the qubit experiences a pure dephasing rate of 2Γsinh^2^(η/2) per photon without frequency shifts. This dissipative interaction is bidirectional but very asymmetric between the qubit and the cavity.

As we show in section S1A, introduction of these two types of dissipative interactions, together with the regular dispersive Hamiltonian, $\frac{\lambda }{2}{\hat{\sigma }}_{z}{\hat{a}}^{†}\hat{a}$ , captures any linear bath–mediated qubit-cavity interaction in the dispersive regime quite generally, assuming the adiabatic approximation holds. Bypassing the intricate details of the bath modes,

the effective model of Eq. 2 allows description of arbitrary quantum dynamics in the qubit-cavity Hilbert space. To experimentally specify the model parameters of our system, we can use a small set of measurements where the cavity is initialized in a coherent state, and its subsequent dynamics can be described by semiclassical mode amplitude and fluctuations. By combining measurements of the photon-induced qubit phase shift and dephasing over a given time window (Fig. 2, A and B) and measurements of the cavity frequencies (Fig. 2, C and D) and decay rates (Fig. 4) for both qubit states, we can obtain six real observables to uniquely determine all the parameters in the master equation (Materials and Methods).

The applied magnetic field *B* provides an in situ tuning knob that allows us to access many distinct instances of the model, each determined independently with parameters plotted in Fig. 3C. How the magnetic field controls each of the model parameters depends on complex details of the intermediary bath modes, which is not a focus of our study. Nevertheless, we observe in Fig. 3C that Γsinhη, λ, and Δc at ±*B* are symmetric. This is the consequence of microscopic symmetry requirements. In a linear system with microreversibility, the Onsager-Casimir relation (*39*) requires that the full scattering matrix *S* satisfy *S*(−*B*) = *S*^T^(*B*). This relation holds separately for each qubit states since our original multimode system dynamics conserves ${\hat{\sigma }}_{z}$ . As a result, the effective complex frequency of the cavity must be symmetric with respect to magnetic fields ±*B* for both qubit states, which leads to the symmetry of λ, Δc, Γsinhη, and κ + Γcoshη (see section S1C for details). Note that an analogous theoretical argument predicts that κ should be symmetric in *B*, which is apparently violated by the data in Fig. 3C and will require further investigation. This might be explained by the presence of other long-lived modes in the system (such as a different cavity mode or a magnon mode) that are weakly coupled to the qubit and off-resonantly excited, which may make the calculation of Γ and thus κ less accurate. We also observed temporal fluctuations in the internal loss rate of the cavity, which may have contributed to this discrepancy.

### Verification of the qubit-cavity dynamics

At this stage, we have used a set of experimental measurements to characterize the parameters of the general master equation model in Eq. 2 that should describe a generic nonreciprocal, dissipative dispersive qubit-cavity interaction. Of course, this extraction of parameters does not by itself show the validity or utility of our model. Now, we use our fully constrained model to make predictions for independent experiments (with different initial states and/or drives) and compare these directly against experimental results.

**Fig. 5.**
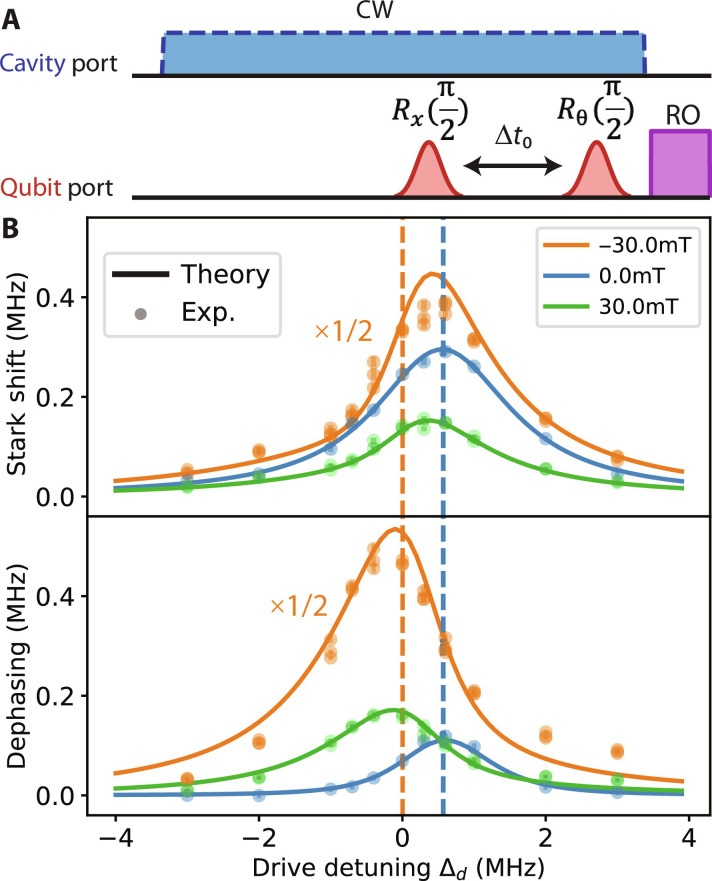
Parameter-free verification of the master equation model with continuous cavity drive. (**A**) The pulse diagram and (**B**) example of the theory prediction (solid line) and experimental result (dot) of qubit Ramsey measurement under steady state CW (5 μs) cavity drive at selective fields (constant drive amplitude across detunings). The Ramsey is performed with a fixed time window at the end of the CW tone. At each CW detuning, the Ramsey data give the Stark shift and dephasing rate. The result at −30 mT is scaled by 1/2 for better visualization. The blue dashed line indicates the cavity frequency Δc at 0 mT, and the 0-mT Stark shift and dephasing peaks correspond well with this frequency, as expected. The orange dashed dot line indicates Δc at ±30 mT. Here, the peak frequency in Stark shift and dephasing rates are distinctly different from each other. See section S2E for more data at different fields.

In the first verification experiment, we investigate the qubit response to continuous wave (CW) cavity drive. Here, we drive the cavity continuously at constant amplitude and varying frequencies and measure the resultant ac Stark shift and photon shot noise–induced dephasing rate on the qubit using a Ramsey sequence (Fig. 5). Note that the cavity is stabilized to a steady state during the Ramsey protocol as opposed to undergoing free decay in the experiments presented in section S2B. The model prediction of the driven system, which can be solved after appending a drive term $\epsilon \left({\hat{a}}^{†}e^{-i\Delta_{d}t}+h.c.\right)$ to the Hamiltonian in Eq. 2, agrees quite well with the experimental data with no free parameters (see section S1E for details). We observe that the Stark shift and dephasing rate often display distinctly different peak frequency (e.g., 30 and −30 mT as shown in Fig. 5B), which is unexpected for a traditional dispersively coupled qubit-cavity system and therefore is a signature of the nonreciprocal interaction in our system. This nontrivial feature in the frequency and line shape is well captured by our model. In comparison, at 0 mT, when the system is close to reciprocal, the Stark shift and decay rate peak at a similar frequency.

**Fig. 6.**
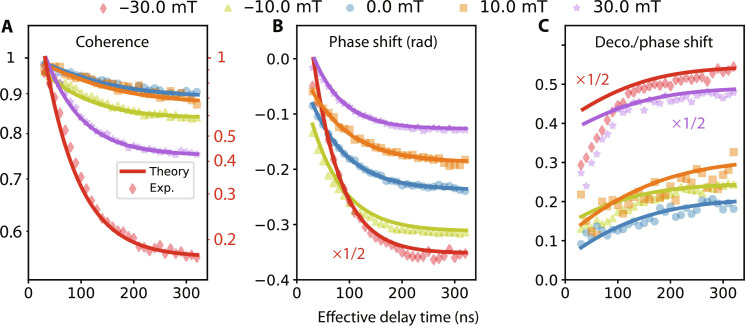
Experiment-theory comparison of the transient dynamics. Time-domain values of (**A**) qubit coherence (ζ), (**B**) accumulated phase shift (ϕ), and (**C**) the ratio between qubit decoherence and phase shift [ln(ζ)/ϕ] of qubit Ramsey measurement results at −30 mT (red), −10 mT (yellow), 0 mT (blue), 10 mT (orange), and 30 mT (purple). The cavity is initialized in a coherent state for all data. The scatter dots are experimental results, and solid lines are theory predictions from the master Eq. 2. The −30-mT data in the coherence panel is plotted according to the red *y* scale on the right side, the −30-mT data in phase shift panel and ±30-mT data in the decoherence/phase shift panel are scaled by 1/2 for better relative visual scaling. Experimental dots start at 30 ns considering the 60-ns qubit pulse right before readout pulse. An ad hoc horizontal shift of 33 and 31 ns has been applied to the theory curve (see text).

In another test of the master equation model, we investigate the time-domain evolution of the qubit in the presence of cavity photons. The experimental protocol is the same as in Fig. 2A, where the cavity is initialized in a coherent state and the qubit is initialized in an equator state. In Fig. 6 (A and B), we show the qubit coherence factor ζ and the cumulative phase shift ϕ as a function of time *t* for a range of external fields. The slopes of the coherence factor (on a log scale) and the phase shift on these plots correspond to the instantaneous photon shot noise dephasing rate and ac Stark shift, and the decrease of slopes over time indicates the continuous decay of cavity photon numbers. A nontrivial feature to be noted is that the dephasing and frequency shift effects decay on slightly different timescales, which can be seen more clearly from their ratio, ln(ζ)/ϕ, which is not a constant over time (Fig. 6C). The time evolution based on the master equation model can be solved as shown in Materials and Methods (Eq. 10) and are plotted as solid curves for comparison. Since the model is applicable on timescales where all fast bath degrees of freedom can be adiabatically eliminated, discrepancies are expected on short timescales. This is especially true at higher magnetic fields when the buffer cavity enclosing the qubit has longer lifetimes (≳20 ns). The model captures the time-domain data well at low fields (e.g., 0 and ±10 mT). At higher fields, we find that the model can describe the temporal evolution reasonably if one allows for an empirical time offset as a fit parameter, which is 33 and 31 ns for ±30 mT in Fig. 6. This ad hoc modification to the model can be understood as some “turn-on” time allowing for the bath (which is not infinitely fast) to reach the quasi-steady state set by the initial condition of the qubit-cavity system. Figure 6C provides a more sensitive test of the model, which correctly captures the time-varying ratio of the dephasing and phase shift effects, although the discrepancies at short times and higher fields become apparent due to the limited separation of timescales.

Last, we apply Eq. 2 to scenarios with non-Gaussian cavity states. Specifically, we consider cavity-qubit dynamics where the cavity is initialized in a single-photon Fock state. Here, we can no longer understand the system via semiclassical equations of motion for mode amplitudes (e.g., as in Eqs. 8 and 11 that we used to characterize the model parameters), but Eq. 2 remains valid and provides direct insights to the nonreciprocal qubit-cavity dynamics. An interesting special case is η = 0, where the dissipator $\Gamma D\left[e^{\frac{i\theta }{2}{\hat{\sigma }}_{z}}\hat{a}\right]$ implements a unitary gate $e^{\frac{i\theta }{2}{\hat{\sigma }}_{z}}$ on the qubit when the photon escapes the cavity. Therefore, if the cavity does not lose the photon via other channels (i.e., κ = 0), then the qubit will receive a deterministic phase shift of θ after *t* ≫ 1/Γ without incurring photon-induced dephasing. A practical application of this deterministic phase shift is microwave single-photon detection as was implemented in (*40*, *41*). These experiments used a conventional circulator to enforce directionality and were understood as the interaction between a qubit and a travelling photon in a transmission line. Our model effectively describes the interaction between the detector qubit and the source cavity of the photons and is further generalized to allow the nonreciprocity of the interaction channel to be varied continuously.

Our experimental platform, aided by the ancilla inside the cavity, allows us to generate single photons and investigate their interaction with the qubit over a range of model parameters by varying magnetic fields. A cavity Fock state |1〉 can be generated by first preparing the ancilla transmon in the |*f*〉 state (its second excited state) and applying a strong drive to induce the four-wave-mixing (FWM) |*f*0〉 − |*g*1〉 sideband transition, which converts the double excitations in the ancilla to a single cavity photon. Experimentally, our FWM transition rate is limited to 0.6 MHz, which, compared to cavity total decay rate of 1.5 to 4 MHz, is not fast enough to instantaneously initialize the cavity in |1〉 before the dissipative process takes place. Nevertheless, in the limit where the ancilla’s |*f*〉 state is perfectly prepared and long-lived, under a sufficiently long FWM drive, there will be one and only one photon generated and emitted from the cavity, effectively realizing single-photon dynamics in our qubit-cavity system.

**Fig. 7.**
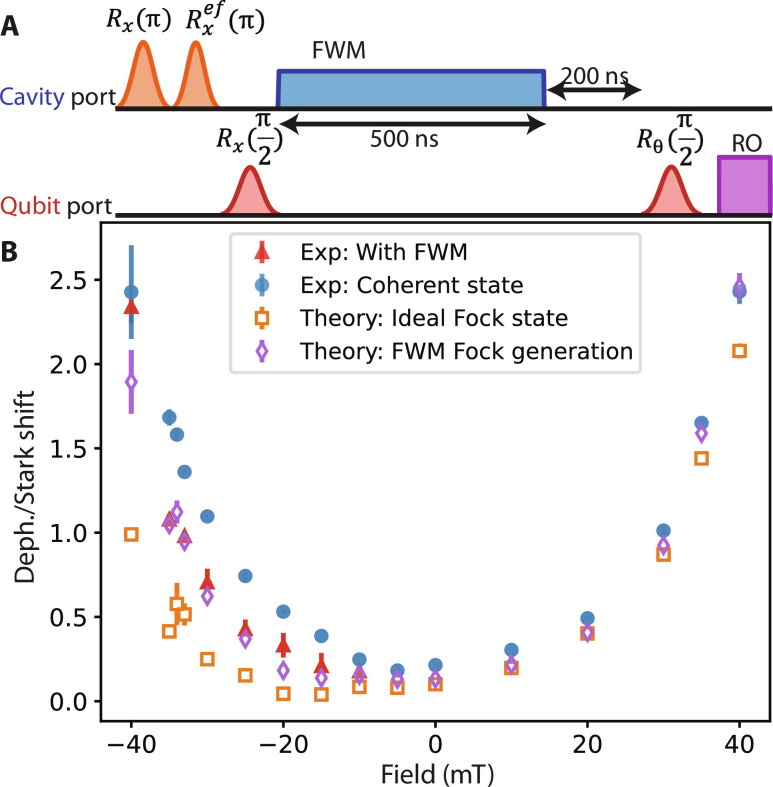
Parameter-free verification of the master equation with single photon generation. (**A**) The pulse diagram of qubit Ramsey experiment in the presence of a single photon generated by an FWM pulse. After initializing the ancilla in |*f*〉, a |*f*0〉 − |*g*1〉 FWM pulse is applied to convert the ancilla |*f*〉 state to a cavity photon. (**B**) The experimental result of the qubit dephasing/Stark shift ratio from the |*f*0〉 − |*g*1〉 FWM measurement (red triangle) and coherent cavity state measurement (blue circle), compared to theory predictions with different initial states: ideal single photon Fock state (orange square), FWM Fock state generation mimicking the experimental setups (purple diamond), which considers the initial thermal population of the ancilla and pulse infidelity thus set ancilla state initializing at 85% in |*f*〉 state, and the ancilla *T*_1_ decay during the FWM pulse.

We implement this approach of single-photon generation with a pulse sequence as shown in Fig. 7A and measure the long-time (*t* = 700 ns) cumulative phase shift ϕ and dephasing ln(ζ) of the qubit similarly as in previous coherent state–based experiments. When the bath modes of the device function approximately like a circulator in the direction from the cavity to the qubit (near *B* ≈ −20 mT), one can expect suppressed qubit dephasing but nonzero phase shift for a single photon. Therefore, the suppressed ratio of ln(ζ)/ϕ is a distinctive signature of the Fock state–qubit interaction compared to a coherent state-qubit interaction. In Fig. 7B, we show that ln(ζ)/ϕ is smaller for the Fock state–induced dynamics for a range of negative fields. (At positive fields, both phase shift and dephasing are too small to be measured accurately in the single photon limit.) The experimentally measured dephasing factor in the Fock state experiment, as reflected in the ln(ζ)/ϕ ratio (red data points), is larger than the Eq. 2 prediction (see section S1F) assuming a perfect initial Fock state. After accounting for limited conversion efficiency (about 89%) of the |*f*0〉 − |*g*1〉 sideband transition in the presence of ancilla *T*_1_ decay and limited fidelity (estimated about 85%) in preparing the ancilla |*f*〉 state, the experimental data agree well with the model prediction. The experiment also illustrates that the ln(ζ)/ϕ ratio for coherent state dynamics is approximately symmetric with respect to magnetic field, in agreement with theoretical predictions based on Onsager-type relations (see section S1D), and this symmetry no longer holds for general quantum dynamics of non-Gaussian states.

## DISCUSSION

In this work, we have realized a dispersive type of nonreciprocal interaction between a qubit and a linear cavity in superconducting circuit QED. This effective interaction, manifested as asymmetric frequency pulls without direct excitation exchange, is mediated by a dissipative bath with broken time reversal symmetry. We introduced a general one-qubit one-cavity master equation model, Eq. 2, which extends the ubiquitous qubit-cavity dispersive interaction in circuit and cavity QED to a dissipative setting and allows simple predictions of qubit-cavity joint dynamics without tackling the complexity of the bath. We verify the efficacy of this master equation model through measurements of the qubit dynamics interacting with continuous cavity drive, initial cavity coherent states, and single-photon states.

While the use of nonreciprocity in superconducting circuits so far has been primarily limited to canonical circulators in peripheral input/output settings, substantial efforts are under way to integrate nonreciprocal elements with the core part of the quantum devices. These studies, for example, range from the development of on-chip superconducting parametric circulators for qubit readout (*9*, *34*, *35*), the use of commercial circulators as directional links between quantum modules (*42*, *43*), to the development of one-way emitters in waveguide QED (*21*, *23*) and the realization of chiral cavity QED for topological many-body physics (*33*, *44*). As bath-mediated nonreciprocity becomes more deeply embedded in the devices, complex interactions between quantum modes and components will arise beyond the dichotomy of direct (capacitive/inductive) coupling and cascaded coupling (via a circulator). To design and to characterize these devices with embedded nonreciprocal elements, it is crucial to have an effective model that are not only general enough to capture the main features and imperfections of nonreciprocity but also simple enough to not invoke the dynamics of the bath. Our work fulfills this need in future engineering and utilization of nonreciprocal circuit QED systems.

Beyond circuit QED, demonstration of dispersive nonreciprocity opens a frontier in the study of nonreciprocity beyond standard scattering-type interactions. For example, making use of the intrinsic connection between those nonreciprocal dynamics and measurement-and-feedforward processes (*25*, *45*), having access to those dynamical elements, could enable realization of passively protected quantum memory (*46*) and even autonomous quantum error correction (*47*). The dispersive nonreciprocity demonstrated here also provides a powerful building block for dissipative quantum simulation. For example, there is immense interest in classical many-body models with kinetic constraints that are inherently directional (*48*–*51*). Our dispersive nonreciprocal interaction provides a route for realizing quantum analogs of such models. More generally, our work thus lays the foundation for investigating and engineering the dispersive type of nonreciprocity, both in the quest for novel dissipative quantum phases of matter and for potential applications in quantum technologies.

## MATERIALS AND METHODS

### Device details

As shown in Fig. 1A, a ϕ-5.58 mm × 5.0 mm single-crystalline YIG cylinder is placed at the center of a copper waveguide Y-junction, with external magnetic fields applied along its height (the [111] orientation of the YIG crystal). The three sides of the Y-junction are connected, respectively, to (i) a narrow-band niobium superconducting cavity (the cavity of interest), which also contains an ancilla transmon for the convenience of cavity state preparation and analysis, (ii) a broadband niobium cavity which functions as a buffer mode and contains the transmon qubit (the qubit of interest), and (iii) a 50-ohm transmission line which is the source of the collective dissipation of the intermediary modes.

The collective spin precession inside the YIG crystal (magnon excitations) is hybridized with the electromagnetic modes in the vicinity of the waveguide Y-junction in a chirality-dependent manner, forming a series of chiral photon-magnon polariton modes (*26*, *27*, *52*). In particular, a pair of near-degenerate polariton modes with zero-field frequency close to 10.8 GHz are primarily responsible for generating an effective dissipative linear coupling between the cavity and the buffer mode. External magnetic fields lift the degeneracy of the polariton mode pair, resulting in clockwise and counterclockwise eigenmodes with mode splitting approximately proportional to magnetic field in the regime below magnetic saturation (see section S2B for more detailed discussion of an ideal toy model of the intermediary mode coupling structure). When the mode splitting approximately matches the loaded loss rate (*B* ≈ ±20 mT for this mode pair), the Y-junction functions approximately like a waveguide circulator with an operating bandwidth of a few hundred megahertz (set by the few hundred megahertz loss rate via the transmission line) near 10.8 GHz (*53*). This device allows us to vary the external magnetic field and hence access coupling channels beyond the special case of the canonical circulator. Note that we apply demagnetization training cycles to suppress the magnetic hysteresis effect before performing experiments at the zero field, thus the hysteresis is negligible. More details of our YIG-cavity device platform and a modeling of the few-mode cavity polariton system without qubits can be found in a previous article (*26*). However, we emphasize that explicit modeling of the lossy intermediary modes based on limited characterization tools (such as the cavity transmission measurements, see fig. S2) does not yield accurate prediction of the qubit-cavity dynamics. This highlights the usefulness of the master equation model Eq. 2 which does not require detailed knowledge.The Hamiltonian and coherence parameters of the cavity and qubit modes are listed in Table 1. We used the TE201 mode of two rectangular cavities for the main cavity mode and the buffer mode, whose frequencies are closely matched to each other using tuning screws and designed to be in the vicinity of the aforementioned Y-junction polariton modes near 10.8 GHz. The frequencies of the qubit and the ancilla are far detuned from cavity modes; hence, their coupling to the dissipative modes is deeply in the dispersive regime. The ancilla is a transmon dispersively coupled to the cavity, so that setting the ancilla transmon in ground or excited state would keep the cavity in different frequencies. Thus, we can read the transmon’s state through regular dispersive readout, by sending a readout pulse at the cavity’s resonance frequency, and checking the response amplitude or phase to readout the ancilla. We have also carried out similar experiments on a second device with similar cavity parameters but different transmon parameters, and the results are included in the Supplementary Materials.

**Table 1.** Experimental parameters for the device used in the measurements. The cavity linewidth is a range rather than fixed value as it is dependent on the field and the qubit state.


Qubit	Frequency	9.141 GHz	χ_0_/2π	5.0 MHz
	Anharmonicity	451 MHz		
	*T*_1_	5.3 μs		
	*T*_2_	2.2 μs		
	*T*_2_ echo	2.7 μs		
Buffer cavity	Frequency	10.808 GHz		
	Linewidth	>5 MHz		
Cavity	Frequency	10.809 GHz	χa/2π	1.1 MHz
	Linewidth	1.5–4.0 MHz		
Ancilla	Frequency	8.277 GHz		
	Anharmonicity	490 MHz		
	*T*_1_	10.2 μs		
	*T*_2_	2.9 μs		
	*T*_2_ echo	3.7 μs		

### General theory of dispersive nonreciprocity

Here, we outline the general theory framework, where one starts from minimal assumptions about the microscopic system and derives the general model in Eq. 2. Detailed derivations can be found in section S1A. The nonreciprocal device studied in this work generally consists of one cavity mode *c*_1_ (mode *a* in the main text), which is coupled to additional waveguide and circulator modes denoted by *c_j_* (*j* = 2,3…, *N*), as well as a qubit dispersively coupled to the waveguide modes. In what follows, we assume that the qubit is strongly coupled to a single waveguide mode *c*_2_, which corresponds to the bare buffer cavity mode used in this work. However, we note that the final result here, i.e., the effective master Eq. 2, also holds in more general cases where the qubit is dispersively coupled to multiple intermediary modes. The total system dynamics can be described by a Lindblad master equation (setting ℏ = 1)

$$\partial_{t}\hat{\rho }=-i\left[{\hat{H}}_{0}+\frac{\omega_{q}}{2}{\hat{\sigma }}_{z}+\frac{\chi_{0}}{2}{\hat{\sigma }}_{z}{\hat{c}}_{2}^{†}{\hat{c}}_{2},\hat{\rho }\right]+L_{\text{diss}}\hat{\rho }$$ (3)

where ${\hat{H}}_{0}$ denotes the Hamiltonian of the coupled linear-mode system, and *L*_diss_ encodes the dissipative dynamics of the total cavity-circulator system. The dynamics of the bosonic modes alone is quadratic, so that we have

$${\hat{H}}_{0}=\sum_{\ell ,m=1}^{N}\;h_{\ell m}{\hat{c}}_{\ell }^{†}{\hat{c}}_{m}$$ (4)

$${ℒ}_{\text{diss}}\hat{\rho }=\sum_{\ell ,m=1}^{n}\;\Gamma_{\ell m}\left({\hat{c}}_{m}\rho {\hat{c}}_{\ell }^{†}-\frac{1}{2}\left\{{\hat{c}}_{\ell }^{†}{\hat{c}}_{m},\hat{\rho }\right\}\right)$$ (5)

Note that the coefficient matrices *h*_ℓ*m*_ and Γ_ℓ*m*_ generally depend on the external field *B*.

We focus on the regime where the waveguide and circulator modes evolve at timescales much faster than the cavity-qubit system of interest, which agrees with the experimental system. In this case, we can integrate out the waveguide and circulator degrees of freedom to obtain an effective description of the cavity-qubit dynamics. This adiabatic elimination can be carried out, e.g., using the standard procedure (*54*) directly with Lindbladians; see also section S1A for an explicit derivation based on quantum Langevin equations of motion and section S1B for an in-depth discussion about how the general model is valid for the experimental systems considered in this work. The final effective quantum master equation generally takes the form of Eq. 2.

### Calibration of cavity photon number

**Fig. 8.**
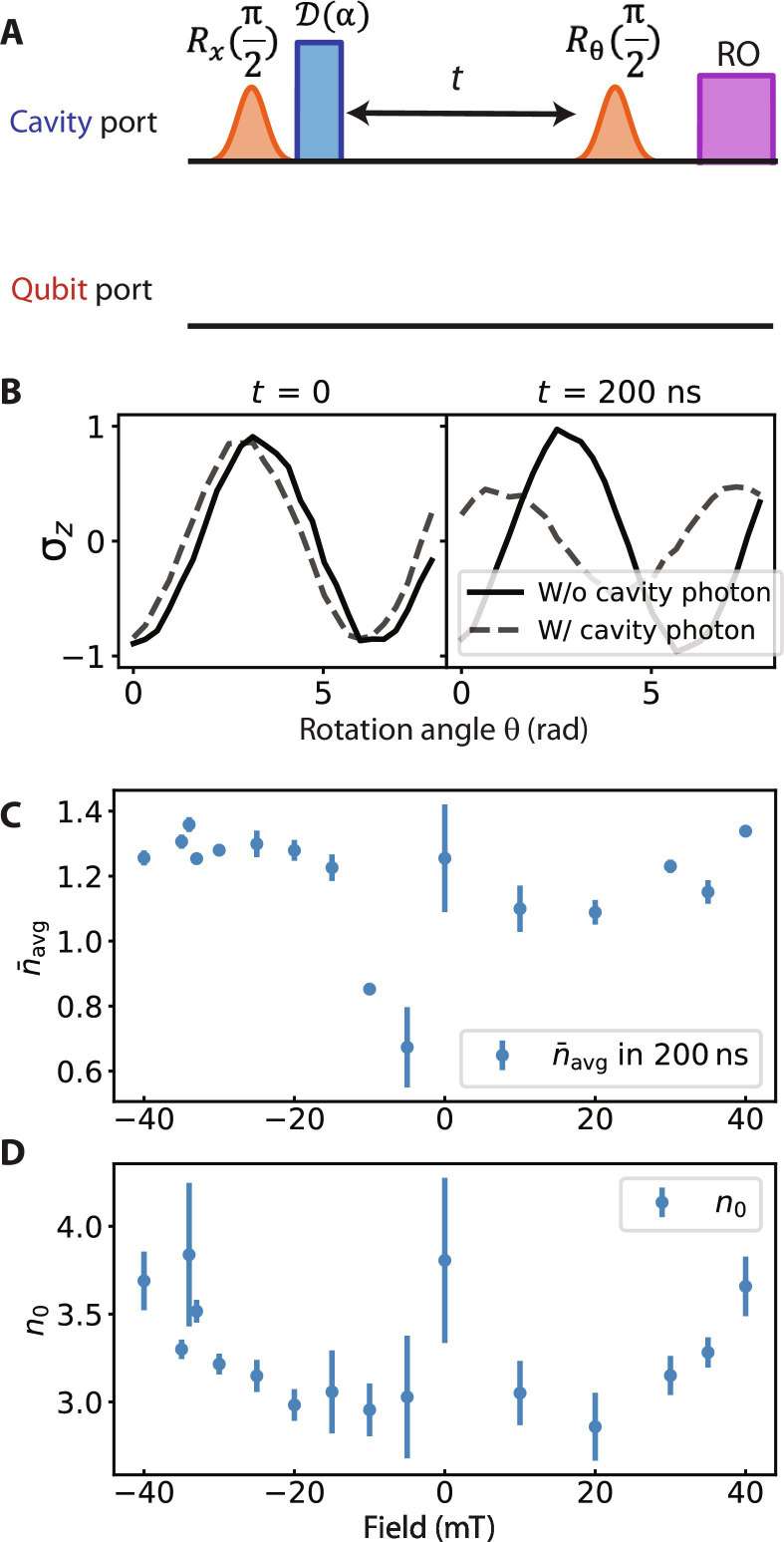
Photon number calibration. (**A**) The pulse diagram and (**B**) the example experimental result of cavity photon number calibration for an ancilla qubit Ramsey measurement under cavity photons. We obtain the ancilla phase evolution by varying the delay time between the cavity pulse and the second ancilla π/2 pulse. The result of the ancilla state against the rotation angle θ is plotted. We obtain the phase and the amplitude from these sinusoidal curves. The solid and dashed lines are for the results without or with cavity photons, respectively, where the phase difference between them is the accumulated phase of ancilla due to cavity photons. (**C**) The averaged cavity photon number during 200 ns, based on the time-averaged frequency shift divided by the dispersive shift χa between the cavity and the ancilla. (**D**) Initial photon number calculated with Eq. 7.

The averaged photon number ${\bar{n}}_{\text{avg}}$ is required for calculating the qubit frequency shift per cavity photon, $\chi_{\text{cq}}=\phi /\left(t\cdot {\bar{n}}_{\text{avg}}\right)$ . To calibrate ${\bar{n}}_{\text{avg}}$ , we measure the ancilla that is directly dispersively coupled to the cavity, where we perform Ramsey experiments on the ancilla with and without cavity photons. By comparing these measurements, we can extract the accumulated extra ancilla phase shift ϕa caused by cavity photons over a finite time window of *t* = 200 ns, as shown in Fig. 8A. Example results of the ancilla state versus rotation angle θ of the second Ramsey π/2 pulse is plotted in Fig. 8B, with the solid and dashed lines representing cases without and with cavity photons, respectively. As ϕ_a_ is proportional to the ${\bar{n}}_{\text{avg}}$ , with the ratio set by dispersive coupling strength χ_a_ between the ancilla and the cavity, $\phi_{a}=\chi_{a}{\bar{n}}_{\text{avg}}t$ , we obtain ${\bar{n}}_{\text{avg}}$ in the 200-ns time window,

$${\bar{n}}_{\text{avg}}=\phi_{a}/\left(\chi_{a}\cdot t\right)$$ (6)

and repeat this protocol for all of the external fields, the result is plotted in Fig. 8C.

The initial photon number ${\bar{n}}_{0}$ is a required quantity for computing the master equation parameters. We can compute its value via the measured cavity decay rate conditioned on the qubit in ground state, κg, as well as the time-averaged cavity photon number ${\bar{n}}_{\text{avg}}$ during the time window *t* (see paragraph above for discussion on its measurement), making use of the following equation

$${\bar{n}}_{0}={\bar{n}}_{\text{avg}}\frac{\kappa_{g}t}{1-e^{-\kappa_{g}t}}$$ (7)

The initial photon number ${\bar{n}}_{0}$ is plotted against varying magnetic fields in Fig. 8D.

### Characterization of system parameters in the master equation model

To motivate our measurement protocol to determine the model parameters, it is worth first discussing the physical meaning of the dynamics generated by Eq. 2: The cavity now experiences a qubit-state–dependent frequency detuning $\omega_{\sigma_{z}}$ and decay rate $\kappa_{\sigma_{z}}$, whereas the qubit undergoes a time-dependent cavity photon–induced phase shift and dephasing effect. It is straightforward to see that the qubit-state–dependent cavity frequency detuning $\omega_{\sigma_{z}}$and decay rate $\kappa_{\sigma_{z}}$ (where σz = ↑, ↓) can be expressed as

$$\omega_{\sigma_{z}}=\Delta_{c}+\frac{\lambda }{2}\sigma_{z},\;\kappa_{\sigma_{z}}=\kappa +\Gamma e^{\eta \sigma_{z}}$$ (8)

Furthermore, the qubit undergoes a time-dependent, cavity photon–induced phase shift and dephasing effect with the cavity initialized in a coherent state (at *t* = 0) in the qubit Ramsey experiment. From Eq. 2, the qubit coherence obeys the equation of motion (see section S1D for derivations)

$$\frac{d\langle {\hat{\sigma }}_{-}\left(t\right)\rangle }{\mathrm{dt}}=\left[-i\lambda +\Gamma \left(e^{i\theta }-\text{cosh}\eta \right)\right]{\bar{a}}_{↑}{\bar{a}}_{↓}^{*}\langle {\hat{\sigma }}_{-}\left(t\right)\rangle$$ (9)

Integrating Eq. 9 over time leads to the following equation relating the qubit phase shift ϕ and decoherence factor lnζ to system parameters, as

$$\begin{array}{c} \text{ln}\frac{\langle {\hat{\sigma }}_{-}\left(t_{f}\right)\rangle }{\langle {\hat{\sigma }}_{-}\left(0\right)\rangle }\equiv (i\phi +\text{ln}\zeta )=\\ {\bar{a}}_{↑}\left(0\right){\bar{a}}_{↓}^{*}\left(0\right)\frac{-i\lambda +\Gamma \left(e^{i\theta }-\text{cosh}\eta \right)}{i\lambda +\kappa +\text{Γcosh}\eta }\times \\ \left(1-e^{-\left(i\lambda +\kappa +\text{Γcosh}\eta \right)t_{f}}\right) \end{array}$$ (10)

where ${\bar{a}}_{↑/↓}\left(0\right)$ are the initial cavity amplitudes conditioning on qubit state. Assuming that ${\bar{a}}_{↑}\left(0\right)\approx {\bar{a}}_{↓}\left(0\right)$ (short drive pulse limit), we can factor out the drive-dependent term ${\bar{a}}_{↑}\left(0\right){\bar{a}}_{↓}^{*}\left(0\right)$ by rescaling the equation with respect to the cavity photon number at *t* = 0, ${\bar{n}}_{0}$ . Taking the long evolution time limit, i.e., when the cavity photon has fully decayed [*t_f_* ≫ 1/(κ + Γ)], Eq. 10 further simplifies to the following

$$\text{ln}\frac{\langle {\hat{\sigma }}_{-}\left(t_{f}\right)\rangle }{\langle {\hat{\sigma }}_{-}\left(0\right)\rangle }≃-{\bar{n}}_{0}\frac{i\lambda +\Gamma \left(\text{cosh}\eta -e^{i\theta }\right)}{i\lambda +\kappa +\text{Γcosh}\eta }$$ (11)

Measurements of the six real observables (i.e., three complex numbers) on the LHS of Eqs. 8 and 10 together provide sufficient constraints to uniquely determine all the parameters in the master equation Eq. 2

$$\Delta_{c}=\left(\omega_{g}+\omega_{e}\right)/2$$ (12)

$$\lambda =\omega_{g}-\omega_{e}$$ (13)

$$\text{Γsinh}\eta =\left(\kappa_{g}-\kappa_{e}\right)/2$$ (14)

$$\kappa +\text{Γcosh}\eta =\left(\kappa_{g}+\kappa_{e}\right)/2$$ (15)

$$\text{Γsin}\theta =\lambda +\text{Im}\;\left[\frac{i\lambda +\left(\kappa_{g}+\kappa_{e}\right)/2}{1-e^{\left[-i\lambda -\left(\kappa_{g}+\kappa_{e}\right)/2\right]t_{f}}}\frac{i\phi +\text{ln}\zeta }{{\bar{n}}_{0}}\right]$$ (16)

$$\Gamma \left(\text{cosh}\eta -\text{cos}\theta \right)=-\frac{1}{2}\text{Re}[\frac{i\lambda +\left(\kappa_{g}+\kappa_{e}\right)/2}{1-e^{-\left[i\lambda +\left(\kappa_{g}+\kappa_{e}\right)/2\right]t_{f}}}\times \frac{i\phi +\text{ln}\zeta }{{\bar{n}}_{0}}]$$ (17)

Note that so far in this derivation of Eqs. 2 and 10, we have neglected intrinsic decoherence of the qubit for simplicity, which, in experiments, is calibrated out by performing differential measurements with or without the cavity drives.

The six experiments used to determine the six parameters are:

1) The qubit Ramsey measurements of its coherence function $\langle {\hat{\sigma }}_{-}\left(t_{f}\right)\rangle$ in the presence of cavity photons, as discussed in section S2B and Fig. 2 (A and B). These measurements give ζ and ϕ. We use data at *t_f_* = 700 ns instead of 200 ns in this analysis as focusing on the long-time integration is expected to provide the largest signal and the best stability.

2) The cavity Ramsey experiments of its frequencies conditioned on both qubit states, as discussed in section S2B and Fig. 2 (C and D), which provide ωg and ωe.

3) The cavity decay rate measurements, as shown and discussed in Fig. 4 and its caption in section S2C, which give κg and κe.

In either type of cavity measurements, it is not possible to perform direct heterodyne detection of cavity photons as it requires larger photon numbers for reasonable measurement time, which will be affected by higher-order spurious nonlinearities. In the cavity Ramsey measurements, to read out the cavity state, we use a method inspired by photon number measurements in the strong dispersive regime (*36*, *37*). Although our system is not in the number-resolved regime, we apply a 200-ns square π-pulse to excite the ancilla (Fig. 2C), whose spectral width is a few times of the cavity-ancilla dispersive shift. The pulse excites the ancilla with decreasing efficiency at increasing photon numbers. Therefore, the measured ancilla |σz〉 provides a monotonic proxy for the cavity photon number (although the relationship is not linear), which is sufficient for an accurate measurement of the free-evolution frequency of the cavity.

In measuring the cavity decay rate, however, it is important to implement a reliable readout scheme that detects the mean cavity photon number in the range of 0.1 to 10 in a linear fashion. To tackle this challenge, we devised a scheme that maps the cavity photon number to the ancilla’s phase shift ϕ over a sliding time window of fixed length (τ = 100 ns, as shown in Fig. 4A), which is measured in a Ramsey sequence of the ancilla. Although the instantaneous cavity photon number changes substantially during the time window τ, the cumulative phase shift ϕa can be used to infer the average photon number during τ, ${\bar{n}}_{\text{avg}}=\frac{\phi_{a}}{{\tau \chi }_{a}}$ , with χa the dispersive shift between the cavity and the ancilla. We find that ${\bar{n}}_{\text{avg}}$ can be fit well to an exponential decay as the Ramsey window slides in time *t* for both the qubit in |*g*〉 and in |*e*〉, which gives the cavity decay rates for the respective cases. In our experiment, we observe that our cavity decay rates fluctuate over the timescale of hours to days, possibly caused by changing configurations of trapped vortices, but the difference, κg − κe, tends to be mostly stable.

## Supplementary Materials

### This PDF file includes:

Sections S1 to S3 Figs. S1 to S14 Table S1
