# Supplementary material for: Dispersive nonreciprocity between a qubit and a cavity
Source: Sci Adv. 2024 Apr 17;10(16):eadj8796. doi: 10.1126/sciadv.adj8796 (PMC11023507; doi:10.1126/sciadv.adj8796)
Supplement: Supplementary file 1 — Sections S1 to S3 Figs. S1 to S14 Table S1 [file sciadv.adj8796_sm.pdf]

Science Advances
AAAS

Supplementary Materials for

Dispersive nonreciprocity between a qubit and a cavity

Ying-Ying Wang *et al.*

Corresponding author: Chen Wang, wangc@umass.edu

Sci. Adv. **10**, eadj8796 (2024)
DOI: 10.1126/sciadv.adj8796

This PDF file includes:

Sections S1 to S3
Figs. S1 to S14
Table S1

Supplementary Materials

1. THEORY

A. Derivation of the effective master equation with a dissipative dispersive-type interaction

In this subsection, we discuss how the effective master equation (Eq. (2)) in the main text can emerge in very general settings involving the cavity mode a, the qubit, and an unspecified number of intermediary modes. We provide a detailed derivation of the effective model starting from minimal assumptions about the microscopic setup. As we show, the effective master equation (2) can describe a wide range of setups with very distinct microscopic mediating modes, yet the cavity-qubit dynamics all converge on a similar simplified form, whose structure could be deduced from general symmetry arguments. It is worth noting that a specialized version of Eq. (2) is discussed in Ref. [25], where the nonlinear dissipator only involves a qubit phase shift instead of the more generic $e^{\frac{i\theta+\eta}{2}\hat{\sigma}_z}$ operator. In contrast, here we show the generalized master equation has broad applicability in dispersive quantum systems exhibiting nonreciprocity.

Below, we use bold text to highlight the essential assumptions underlying the theoretical derivation. In Sec. 1 B, we will discuss how the experimental systems studied in the main text are well described by the theory model presented here. However, we stress that the results in this subsection have general applicability that is not contingent on details of the intermediary modes.

Let us consider a multimode setup (see Fig. 1) consisting of one cavity mode a and a qubit, as well as additional waveguide and circulator modes evolving at timescales much faster than the cavity-qubit system. We assume a general situation where **the waveguide and circulator eigenmodes all have resonances that are far detuned from the cavity-qubit system, as well as decay rates that are much greater than rates characterizing the dynamics of the cavity-qubit system of interest**. In what follows we denote the cavity mode as c_1, and take the cavity and qubit frequencies to be ω_1 and ω_q. Their respective lowering operators are $\hat{c}_1$ and $\hat{\sigma}_-$. We also denote the bosonic waveguide and circulator modes by modes c_j with intrinsic frequencies ω_j ($j = 2, 3 \ldots, N$).

We focus on the regime where **the qubit is only strongly and dispersively coupled to a single waveguide mode, which we call mode** c_2 (frequency ω_2), so that our starting microscopic model can be generally written as

$$\partial_t \hat{\rho} = -i\left[\hat{H}_0 + \frac{\omega_q}{2}\hat{\sigma}_z + \frac{\chi_0}{2}\hat{\sigma}_z\hat{c}_2^\dagger\hat{c}_2, \hat{\rho}\right] + \mathcal{L}_{\rm diss}\hat{\rho}. \quad \text{(S1)}$$

Here, $\hat{H}_0$ and $\mathcal{L}_{\rm diss}$ describe the intrinsic Hamiltonian and dissipative dynamics of the waveguide and circulator modes, and χ_0 denotes the dispersive coupling strength between the qubit and mode c_2. Throughout our discussion, we assume **the dynamics of the bosonic modes alone is quadratic**, so that we have (note that by definition $h_{\ell\ell}^{(0)} = \omega_\ell$)

$$\hat{H}_0 = \sum_{\ell,m=1}^{N} h_{\ell m}^{(0)}\hat{c}_\ell^\dagger\hat{c}_m, \quad \text{(S2)}$$

$$\mathcal{L}_{\rm diss}\hat{\rho} = \sum_{\ell,m=1}^{N}\Gamma_{\ell m}\left(\hat{c}_m\rho\hat{c}_\ell^\dagger - \frac{1}{2}\left\{\hat{c}_\ell^\dagger\hat{c}_m, \hat{\rho}\right\}\right). \quad \text{(S3)}$$

Here, we assume that **thermal excitations of the bosonic modes are negligible**. For systems with an external magnetic field B, the system parameters $h_{\ell m}^{(0)}$ and $\Gamma_{\ell m}$ will also depend on the external field B. For convenience, we omit this parametric B dependence when it does not cause confusion.

Note that the dispersive χ_0 interaction in Eq. (S1) can emerge from a microscopic Jaynes-Cummings interaction between the qubit and c_2

$$\hat{H}_{\rm JC} = g\left(\hat{c}_2^\dagger\hat{\sigma}_- + \hat{c}_2\hat{\sigma}_+\right). \quad \text{(S4)}$$

One needs to consider the limit where the detuning $\Delta_{\rm JC} \equiv \omega_2 - \omega_q \gg |g|$, as well as $\Delta_{\rm JC} \gg |h_{lm}|$. The latter condition allows us to safely neglect the hybridizations h_{lm} when deriving the dispersive coupling χ_0 from $\hat{H}_{\rm JC}$ via a standard Schrieffer-Wolff transformation (see, e.g., [55]). In the case of stronger hybridizations h_{lm}, one will obtain a larger set of starting dispersive couplings in Eq. (S1) between the qubit and multiple internal modes. The general form of the master equation derived below still holds in this case (though the specific expressions for the exact effective parameters in Eqs. (S21) and (S24) would be modified). We focus on the simpler case of a single starting dispersive coupling for clarity.

Next, recall our starting assumption that all the intrinsic waveguide and circulator dynamics (i.e., their resonances and linewidths) are much faster than that of the cavity mode c_1 and qubit. This motivates us to adiabatically eliminate the dynamics of the intermediary modes. We first transform to a rotating frame via $\hat{\rho}(t) \to \hat{\rho}'(t) = \hat{U}_r(t)\hat{\rho}(t)\hat{U}_r^\dagger(t)$, with $\hat{U}_r(t) = e^{i(\frac{\omega_q}{2}\hat{\sigma}_z + \omega_r\sum_\ell \hat{c}_\ell^\dagger\hat{c}_\ell)t}$. Here, all the bosonic modes are rotated by a fixed reference frequency ω_r, which is chosen to be close to the cavity c_1 eigenmode frequency (i.e., the bare c_1 resonance ω_1 plus any Stark shifts induced by the mediating modes) compared with all other resonances in the system. In this new frame, the total system dynamics can be described by

$$\partial_t\hat{\rho} = -i\left[\hat{H}_0' + \frac{\chi_0}{2}\hat{\sigma}_z\hat{c}_2^\dagger\hat{c}_2, \hat{\rho}\right] + \mathcal{L}_{\rm diss}\hat{\rho}, \quad \text{(S5)}$$

$$\hat{H}_0' = \sum_{\ell,m=1}^{N} h_{\ell m}\hat{c}_\ell^\dagger\hat{c}_m, \quad \text{(S6)}$$

with $\hat{H}_0' = \sum_{\ell,m=1}^{N} h_{\ell m}\hat{c}_\ell^\dagger\hat{c}_m$, and $h_{\ell m} = h_{\ell m}^{(0)} - \delta_{\ell m}\omega_r$.

In what follows, we focus on the case where it is valid to adiabatically eliminate the waveguide and circulator modes, i.e. modes c_j with $j > 1$, from the total system. In this regime, one can derive an effective Markovian master equation involving only the cavity mode c_1 and the qubit, which

can be generally written as

$$\partial_t \hat{\rho} = -i[\hat{H}_{\rm eff}, \hat{\rho}] + \mathcal{L}_{\rm diss}^{(\rm eff)} \hat{\rho}. \tag{S7}$$

We now carry out this adiabatic elimination explicitly. While this can be done using a variety of approaches (see, e.g., [54]), here we use an intuitive method based on stochastic equations of motion in the Heisenberg picture. More specifically, the quantum Langevin equations of motion (EOM) for the bosonic modes and the qubit in the rotating frame of Eq. (S1) are given by (assuming Einstein's sum rule hereafter)

$$i\partial_t \hat{c}_\ell = \left(h_{\ell m} + \frac{\chi_0}{2}\delta_{\ell 2}\delta_{m2}\hat{\sigma}_z - \frac{i}{2}\Gamma_{\ell m} \right) \hat{c}_m - i\hat{\xi}_{\ell,\rm in}, \tag{S8}$$

$$i\partial_t \hat{\sigma}_- = \chi_0 \hat{c}_2^\dagger \hat{c}_2 \hat{\sigma}_-, \tag{S9}$$

where $\hat{\xi}_{\ell,\rm in}(t)$ denote quantum input noise operators (incorporating the coefficients characterizing dissipative dynamics in Eq. (S3)) with $\langle \xi_{\ell,\rm in}^\dagger(t)\xi_{m,\rm in}(t')\rangle = 0$ and $\langle \xi_{\ell,\rm in}(t)\xi_{m,\rm in}^\dagger(t')\rangle = \Gamma_{\ell m}\delta(t-t')$. As mentioned, we can integrate out the dynamics of the waveguide and circulator modes if following conditions hold

$$\forall \ell > 1, \text{ and } m \neq j : \Gamma_{\ell\ell} \gg |h_{mj} - \frac{i}{2}\Gamma_{mj}|. \tag{S10}$$

In this limit, the dynamics of modes c_j with $j > 1$ quickly relaxes to a stationary state at timescale much faster than typical dynamics of cavity c_1 and the qubit. Thus, as far as the cavity-qubit system is concerned, we can set $\partial_t \hat{c}_\ell = 0$ for $\ell > 1$, so that Eqs. (S8) simplify to

$$i\delta_{\ell 1}\partial_t \hat{c}_\ell = \left(h_{\ell m} + \frac{\chi_0}{2}\delta_{\ell 2}\delta_{m2}\hat{\sigma}_z - \frac{i}{2}\Gamma_{\ell m} \right) \hat{c}_m - i\hat{\xi}_{\ell,\rm in}. \tag{S11}$$

Note that Eq. (S10) provides a sufficient condition for this approximation to be valid; however, the validity of the adiabatic elimination procedure extends far beyond the specific regime where all the bare intermediary modes have large decay rates. For example, if some of the intermediary modes have large detuning satisfying $h_{\ell\ell} \gg |h_{mj} - \frac{i}{2}\Gamma_{mj}|$ with $\ell > 2$ and $m \neq j$, then those fast oscillating modes can also be safely integrated out. Essentially, as long as the dynamical eigenmodes of the intermediary structure decay faster than their interaction with the cavity-qubit system (i.e., eigenvalues of $\mathcal{H}$ in Eq. (S12) all have a sufficiently large imaginary part), then Eq. (S11) holds to a good approximation.

Solving Eqs. (S8) for $\ell > 1$, and substituting the solutions into the EOM for the cavity (Eq. (S8) with $\ell = 1$) and the qubit, we can compute an effective set of EOMs of the cavity-qubit system, which can be uniquely mapped to a corresponding Markovian master equation in Eq. (S7). We perform this derivation explicitly below. To simplify notations, let us first introduce the standard linear response susceptibilities (or equivalently, retarded Green's functions) $G_{\ell m}[\omega] \equiv -i\int_0^{+\infty} \langle [\hat{c}_\ell(t), \hat{c}_m^\dagger(0)] \rangle e^{i\omega t} dt$ of the linear part of the bosonic dynamics (i.e. setting $\chi_0 = 0$ in Eq. (S1)), which are related to the original Hamiltonian and dissipator parameters as (see also Eq. (S41))

$$G_{\ell m}[\omega] = \left[(\omega - \mathcal{H})^{-1} \right]_{\ell m}, \quad \mathcal{H} = \mathbf{H} - \frac{i}{2}\mathbf{\Gamma}. \tag{S12}$$

Here, we use boldface letters $\mathbf{H}$ and $\mathbf{\Gamma}$ to denote the Hermitian matrices formed by Hamiltonian (Eq. (S6)) and dissipator (Eq. (S3)) coefficients, respectively. Note that the dynamics of the system can now be fully captured via a single non-Hermitian matrix $\mathcal{H}$. We also define shorthand notations for zero-frequency linear response susceptibilities, as well as the determinant of the zero-frequency susceptibility submatrix of modes c_1 and c_2, as

$$G_{\ell m}^{(0)} \equiv G_{\ell m}[\omega = 0], \tag{S13}$$

$$\det G_{\{1,2\}}^{(0)} \equiv G_{11}^{(0)} G_{22}^{(0)} - G_{12}^{(0)} G_{21}^{(0)}. \tag{S14}$$

We now seek to solve Eq. (S11), which can be equivalently rewritten as

$$-\left(h_{\ell m} - \frac{i}{2}\Gamma_{\ell m} \right) \hat{c}_m = -i\delta_{\ell 1}\partial_t \hat{c}_\ell + \frac{\chi_0}{2}\delta_{\ell 2}\hat{\sigma}_z \hat{c}_2 - i\hat{\xi}_{\ell,\rm in}, \tag{S15}$$

so that inverting the dynamical matrix on the left hand side, and noting that $(\mathbf{H} - i\mathbf{\Gamma}/2)^{-1} = \mathcal{H}^{-1} = -\mathbf{G^{(0)}}$, we obtain

$$\hat{c}_\ell = -G_{\ell 1}^{(0)} \cdot i\partial_t \hat{c}_1 + \frac{\chi_0}{2} G_{\ell 2}^{(0)} \hat{\sigma}_z \hat{c}_2 - iG_{\ell m}^{(0)} \hat{\xi}_{m,\rm in}. \tag{S16}$$

Specifically, we consider the $\ell = 1$ and 2 cases of Eqs. (S16), which are given by

$$\hat{c}_1 = -G_{11}^{(0)} \cdot i\partial_t \hat{c}_1 + \frac{\chi_0}{2} G_{12}^{(0)} \hat{\sigma}_z \hat{c}_2 - iG_{1m}^{(0)} \hat{\xi}_{m,\rm in}, \tag{S17}$$

$$\hat{c}_2 = -G_{21}^{(0)} \cdot i\partial_t \hat{c}_1 + \frac{\chi_0}{2} G_{22}^{(0)} \hat{\sigma}_z \hat{c}_2 - iG_{2m}^{(0)} \hat{\xi}_{m,\rm in}, \tag{S18}$$

so that with a few lines of algebra, we can express $\hat{c}_2$ as well as the time derivative $i\partial_t \hat{c}_1$ in terms of only operator $\hat{c}_1$ and the input noise operators $\hat{\xi}_{m,\rm in}$, as

$$i\partial_t \hat{c}_1 = \mathcal{E}_{\sigma_z} \Big[\hat{c}_1 + iG_{1m}^{(0)} \hat{\xi}_{m,\rm in} + i\frac{\chi_0}{2} G_{12}^{(0)} \hat{\sigma}_z (1 - \frac{\chi_0}{2} G_{22}^{(0)} \hat{\sigma}_z)^{-1} G_{2m}^{(0)} \hat{\xi}_{m,\rm in} \Big], \tag{S19}$$

$$\Big[G_{11}^{(0)} - \frac{\chi_0}{2}\hat{\sigma}_z \det G_{\{1,2\}}^{(0)} \Big] \hat{c}_2 = G_{21}^{(0)} \hat{c}_1 - iG_{11}^{(0)} G_{2m}^{(0)} \hat{\xi}_{m,\rm in} + iG_{21}^{(0)} G_{1m}^{(0)} \hat{\xi}_{m,\rm in}. \tag{S20}$$

Since dynamics generated by Eq. (S1) conserves qubit Pauli operator $\hat{\sigma}_z$, we can treat it in the above derivation as if it were a number. Thus, we substitute the operator $\hat{\sigma}_z$ with σ_z whenever it can be viewed as a number parametrizing the cavity dynamics. The effective qubit-state-dependent self-energy $\mathcal{E}_{\sigma_z}$ of the cavity mode c_1 can be derived as

$$\mathcal{E}_{\sigma_z} = -\Big[G_{11}^{(0)} + \frac{\chi_0}{2} G_{12}^{(0)} \sigma_z (1 - \frac{\chi_0}{2} G_{22}^{(0)} \sigma_z)^{-1} G_{21}^{(0)} \Big]^{-1} = -\frac{1 - \frac{\chi_0}{2} G_{22}^{(0)} \sigma_z}{G_{11}^{(0)} - \frac{\chi_0}{2}\sigma_z \det G_{\{1,2\}}^{(0)}}. \tag{S21}$$

Further, substituting Eq. (S20) into Eq. (S9), we also obtain the effective equation of motion of the qubit operator

$$i\partial_t\hat{\sigma}_- = \chi_0\hat{c}_2^\dagger\hat{\sigma}_-\hat{c}_2 \tag{S22}$$

$$=\Lambda\hat{c}_1^\dagger\hat{\sigma}_-\hat{c}_1 - i\Lambda\left[\hat{c}_1^\dagger\hat{\sigma}_-\hat{\Xi}_{\text{in}} - \hat{\Xi}_{\text{in}}^\dagger\hat{\sigma}_-\hat{c}_1\right], \tag{S23}$$

where we define the effective qubit-cavity coupling coefficient Λ and the effective noise operator $\hat{\Xi}_{\text{in}}$ as

$$\Lambda = \frac{\chi_0\left|G_{21}^{(0)}\right|^2}{\left\{G_{11}^{(0)} - \frac{\chi_0}{2}\det G_{\{1,2\}}^{(0)}\right\}\left\{G_{11}^{(0)*} + \frac{\chi_0}{2}\det G_{\{1,2\}}^{(0)*}\right\}}, \tag{S24}$$

$$\hat{\Xi}_{\text{in}} = (G_{21}^{(0)})^{-1}(G_{11}^{(0)}G_{2m}^{(0)}\hat{\xi}_{m,\text{in}} - G_{21}^{(0)}G_{1m}^{(0)}\hat{\xi}_{m,\text{in}}). \tag{S25}$$

In deriving Eq. (S23), we make use of the fact that the quantum input noise operator satisfies $\langle\hat{\Xi}_{\text{in}}^\dagger\hat{\Xi}_{\text{in}}\rangle = 0$. The reader with a keen eye may also notice that the right hand side of Eq. (S22) has a specific operator ordering; this is done to simplify the noise terms in Eq. (S23) such that it exhibits an explicit correspondence with the noise term generated by the dissipator in a master equation, but the validity of our derivations does not rely on any particular choice of ordering in the derivation.

In the following discussion, we relabel the cavity mode c_1 to be mode a, in accordance with the main text discussion. For the derivation below, it is convenient to rewrite the input noise operators in Eqs. (S19) and (S23) as

$$i\partial_t\hat{a} =\mathcal{E}_{\sigma_z}\hat{a} - i\sqrt{\kappa}\hat{\xi}_{\text{in}} - i\sqrt{\Gamma}e^{\frac{-i\theta+\eta}{2}\hat{\sigma}_z}\hat{\eta}_{\text{in}}, \tag{S26}$$

$$i\partial_t\hat{\sigma}_- =\Lambda\hat{a}^\dagger\hat{a}\hat{\sigma}_- + 2i\sqrt{\Gamma}(\sinh\frac{i\theta+\eta}{2})\hat{\eta}_{\text{in}}^\dagger\hat{\sigma}_-\hat{a} - 2i\sqrt{\Gamma}(\sinh\frac{-i\theta+\eta}{2})\hat{a}^\dagger\hat{\sigma}_-\hat{\eta}_{\text{in}}, \tag{S27}$$

where $\hat{\xi}_{\text{in}}$ and $\hat{\eta}_{\text{in}}$ are normalized Markovian quantum input noise operators, so that $\langle\hat{\xi}_{\text{in}}^\dagger(t)\hat{\xi}_{\text{in}}(t')\rangle = 0$ and $\langle\hat{\xi}_{\text{in}}(t)\hat{\xi}_{\text{in}}^\dagger(t')\rangle = \delta(t-t')$ (similarly for $\hat{\eta}_{\text{in}}$). The noise operators can be determined using the following equations

$$\sqrt{\kappa}\hat{\xi}_{\text{in}} + \sqrt{\Gamma}e^{\frac{-i\theta+\eta}{2}\hat{\sigma}_z}\hat{\eta}_{\text{in}} =\frac{1}{G_{11}^{(0)}}\left(G_{1m}^{(0)}\hat{\xi}_{m,\text{in}} + \frac{\frac{\chi_0}{2}G_{12}^{(0)}G_{21}^{(0)}\hat{\sigma}_z}{G_{11}^{(0)} - \frac{\chi_0}{2}\det G_{\{1,2\}}^{(0)}\hat{\sigma}_z}\hat{\Xi}_{\text{in}}\right), \tag{S28}$$

$$2i\sqrt{\Gamma}(\sinh\frac{-i\theta+\eta}{2})\hat{\eta}_{\text{in}} = \Lambda\hat{\Xi}_{\text{in}}. \tag{S29}$$

One can show that the quantum Langevin equations in Eqs. (S26) generally correspond to the Lindblad master equation of the form Eq. (S7), with effective Hamiltonian $\hat{H}_{\text{eff}}$ and dissipative dynamics $\mathcal{L}_{\text{diss}}^{(\text{eff})}$ given by

$$\hat{H}_{\text{eff}} = \Delta_c\hat{a}^\dagger\hat{a} + \frac{\lambda}{2}\hat{\sigma}_z\hat{a}^\dagger\hat{a}, \tag{S30}$$

$$\mathcal{L}_{\text{diss}}^{(\text{eff})} = \kappa\mathcal{D}\left[\hat{a}\right] + \Gamma\mathcal{D}\left[e^{\frac{i\theta+\eta}{2}\hat{\sigma}_z}\hat{a}\right]. \tag{S31}$$

We remark that the effective model preserves symmetry and dynamical features of the original multimode problem: the qubit Pauli operator $\hat{\sigma}_z$ is conserved exactly, and the dynamics of cavity and circulator modes conditioned on qubit $\hat{\sigma}_z$ eigenstates are fully quadratic. The effective parameters in Eq. (S30,S31) can be computed via the equations below

$$\mathcal{E}_{\sigma_z} =\Delta_c + \frac{\lambda}{2}\sigma_z - i\frac{\kappa+\Gamma e^{\eta\sigma_z}}{2}, \tag{S32}$$

$$\Lambda =\lambda + i\Gamma(e^{i\theta} - \cosh\eta). \tag{S33}$$

All individual effective parameters can thus be explicitly derived by combining Eqs. (S32) and (S33) with Eq. (S21) and (S24). Note that Eq. (S21) can be rewritten as

$$\mathcal{E}_{\sigma_z} = -\frac{G_{11}^{(0)} - \left(\frac{\chi_0}{2}\right)^2G_{22}^{(0)}\det G_{\{1,2\}}^{(0)} - \frac{\chi_0}{2}G_{12}^{(0)}G_{21}^{(0)}\sigma_z}{\left(G_{11}^{(0)}\right)^2 - \left(\frac{\chi_0}{2}\sigma_z\det G_{\{1,2\}}^{(0)}\right)^2}, \tag{S34}$$

we thus obtain

$$\Delta_c = -\text{Re}\left[\frac{G_{11}^{(0)} - \left(\frac{\chi_0}{2}\right)^2G_{22}^{(0)}\det G_{\{1,2\}}^{(0)}}{\left(G_{11}^{(0)}\right)^2 - \left(\frac{\chi_0}{2}\det G_{\{1,2\}}^{(0)}\right)^2}\right], \tag{S35a}$$

$$\lambda = \chi_0\text{Re}\left[\frac{G_{12}^{(0)}G_{21}^{(0)}}{\left(G_{11}^{(0)}\right)^2 - \left(\frac{\chi_0}{2}\det G_{\{1,2\}}^{(0)}\right)^2}\right], \tag{S35b}$$

$$\Gamma\sinh\eta = -\chi_0\text{Im}\left[\frac{G_{12}^{(0)}G_{21}^{(0)}}{\left(G_{11}^{(0)}\right)^2 - \left(\frac{\chi_0}{2}\det G_{\{1,2\}}^{(0)}\right)^2}\right], \tag{S35c}$$

as well as the following expression for the averaged cavity decay rate over two possible qubit states

$$\kappa + \Gamma\cosh\eta = 2\text{Im}\left[\frac{G_{11}^{(0)} - \left(\frac{\chi_0}{2}\right)^2G_{22}^{(0)}\det G_{\{1,2\}}^{(0)}}{\left(G_{11}^{(0)}\right)^2 - \left(\frac{\chi_0}{2}\det G_{\{1,2\}}^{(0)}\right)^2}\right]. \tag{S36}$$

Comparing Eq. (S33) with Eq. (S24), we further have

$$\lambda - \Gamma\sin\theta = \frac{\chi_0\left|G_{21}^{(0)}\right|^2\left(\left|G_{11}^{(0)}\right|^2 - \left|\frac{\chi_0}{2}\det G_{\{1,2\}}^{(0)}\right|^2\right)}{\left|\left(G_{11}^{(0)}\right)^2 - \left(\frac{\chi_0}{2}\det G_{\{1,2\}}^{(0)}\right)^2\right|^2}, \tag{S37a}$$

$$\Gamma(\cos\theta - \cosh\eta) = \frac{\frac{\chi_0^2}{2}\left|G_{21}^{(0)}\right|^2\text{Im}\left(G_{11}^{(0)*}\det G_{\{1,2\}}^{(0)}\right)}{\left|\left(G_{11}^{(0)}\right)^2 - \left(\frac{\chi_0}{2}\det G_{\{1,2\}}^{(0)}\right)^2\right|^2}, \tag{S37b}$$

so that substituting Eq. (S35b) into Eq. (S37a), we can explicitly express $\Gamma\sin\theta$ in terms of the microscopic system parameters. Making use of the following identity relation of the rates involving Γ

$$\Gamma(\cos\theta+\cosh\eta)=\frac{\Gamma^2(\cos^2\theta-\cosh^2\eta)}{\Gamma(\cos\theta-\cosh\eta)}=\frac{-(\Gamma\sin\theta)^2-(\Gamma\sinh\eta)^2}{\Gamma(\cos\theta-\cosh\eta)},\qquad(\text{S38})$$

and combing the above equation with Eqs. (S35c), (S36), (S37a), and (S37b), we can solve for the remaining decay rates κ and Γ explicitly.

B. Application of the theory model in Sec. 1 A to the experimental systems in the main text

The theory presented in Sec. 1 A can be generally applicable to a large class of dispersive nonreciprocal systems, whose validity does not rely on details of the underlying mediating modes. Still, the theory does make a few assumptions about the nature of the microscopic environment for the qubit-cavity system. Here, we provide a detailed justification for the validity of those assumptions in our experiment.

- All mediating modes have negligible thermal excitation. This can be verified by an estimate of the steady-state cavity photon (without any driving of the cavity modes). From the dephasing time of the qubit and the ancilla, we can place an upper bound to the thermal occupancy of the cavity and buffer cavity modes at 1% level.
- The qubit is in the dispersive limit. In the experiment, the qubit at 9.1 GHz is Purcell protected by a buffer cavity mode at 10.8 GHz and does not have direct interaction with the rest of system otherwise. The relevant bandwidth of bath modes participating in photon transport in the experiment does not exceed a few hundred MHz (around the 10.8 GHz cavity frequency), hence the qubit is deeply detuned in the dispersive regime.
- The qubit is only dispersively coupled to a single waveguide mode. This is well motivated for the experimental systems considered in this work, as the qubit only has direct interaction with the buffer cavity mode discussed above.
- All the mediating modes are evolving much faster than the cavity-qubit system such that they can be adiabatically eliminated. Experimentally, the lack of sharp resonance features in spectroscopy except for the cavity mode (and the buffer cavity mode at high fields) confirms that the intermediary modes have much larger linewidths and the adiabatic approximation is well justified.

It is also worth commenting on the physical meaning of the Δ_c parameter in relation to experimental parameters. In theory, this denotes the effective cavity eigenfrequency defined with respect to some reference frequency ω_r. In experiment, the microwave pulse frequency used to initialize cavity coherent states provides a natural reference frequency, as it is close (within several MHz) to the cavity resonance. We fix ω_r throughout the experiment across different fields so that the field dependence of the cavity frequency is reflected in the Δ_c parameter.

C. Proof of the generalized Onsager-type symmetry relations

In standard linear systems, the microscopic time reversal symmetry (TRS) naturally imposes a constraint on the system's scattering matrix, which is also known as the Onsager-Casimir reciprocity relations [39] in the presence of magnetic fields. Here, we show such reciprocity relations can be generalized to nonlinear systems with dispersive couplings.

For reference, let us first review standard Onsager-Casimir reciprocity relations [39]. In terms of linear response susceptibilities $G_{\ell m}[\omega]\equiv -i\int_0^{+\infty}\left\langle\left[\hat{c}_\ell(t),\hat{c}_m^\dagger(0)\right]\right\rangle e^{i\omega t}dt$, the Onsager-Casimir relations can be written in the frequency space as

$$G_{\ell m}[\omega;B]=G_{m\ell}[\omega;-B].\qquad(\text{S39})$$

For linear systems (e.g. setting $\chi_0=0$ in Eq. (S1)), the constraints in Eq. (S39) are equivalent to the corresponding symmetry conditions on the Hamiltonian and dissipator matrices. To see this, we can consider the Langevin equations of motion of cavity and circulator modes, which is given by

$$i\partial_t\hat{c}_\ell=\left(h_{\ell m}-\frac{i}{2}\Gamma_{\ell m}\right)\hat{c}_m+i\hat{\xi}_{\ell,\mathrm{in}},\qquad(\text{S40})$$

from which we can explicitly write the linear response susceptibilities as

$$G_{\ell m}[\omega]=\left[(\omega-\mathcal{H})^{-1}\right]_{\ell m}=\left[\left(\omega-\mathbf{H}+\frac{i}{2}\mathbf{\Gamma}\right)^{-1}\right]_{\ell m}.\qquad(\text{S41})$$

Here, the non-Hermitian matrix $\mathcal{H}=\mathbf{H}-\frac{i}{2}\mathbf{\Gamma}$ incorporates effects from both the Hamiltonian $\mathbf{H}$ and the dissipator $\mathbf{\Gamma}$ matrices. From Eq. (S41), one can show the constraints in Eq. (S39) is equivalent to the following conditions on the Hamiltonian and dissipator matrices, as

$$\mathbf{H}(B)=[\mathbf{H}(-B)]^T,\qquad(\text{S42})$$

$$\mathbf{\Gamma}(B)=[\mathbf{\Gamma}(-B)]^T.\qquad(\text{S43})$$

From Onsager-type reciprocity relations on linear response functions in Eq. (S39), one can show that the effective cavity a mode self-energy $\mathcal{E}_{\sigma_z}$ also needs to satisfy the corresponding reciprocity relation, as

$$\mathcal{E}_{\sigma_z}(B)=\mathcal{E}_{\sigma_z}(-B).\qquad(\text{S44})$$

From definition of $\mathcal{E}_{\sigma_z}$ in Eq. (S32), we can further show that the following (combinations of) effective quantum master equation parameters must also be symmetric under $B \to -B$:

$$\Delta_c,\ \lambda,\ \Gamma \sinh \eta,\ \kappa + \Gamma \cosh \eta. \tag{S45}$$

We note that the symmetry constraint on $\mathcal{E}_{\sigma_z}$ can be intuitively viewed as a specific 1-mode case of the more general time reversal symmetry of the dynamical matrix of a linear system: effectively, it can be attributed to the fact that eigenvalues of the non-Hermitian dynamical matrix $\mathcal{H}$ are invariant under time reversal. In contrast, the cavity-to-qubit Λ is directly related to the squared norm of response function $|G_{21}^{(0)}|^2 = |G_{21}[\omega = 0]|^2$ from cavity modes 1 to 2. As such, Λ also encodes information about the eigenvector of $\mathcal{H}$, and hence can generally be asymmetric under time reversal. As a result, the corresponding effective model parameters $\Gamma \sin \theta$ and $\Gamma \cos \theta$ are also in general asymmetric when flipping the sign of the magnetic field B.

D. Solution to the effective quantum master equation with cavity drives

In the main text, we present comparisons between theoretical predictions of the effective master equation (2) (i.e. Eq. (S7)), and experimental measurements of time evolution of the qubit coherence function $\langle \hat{\sigma}_-(t) \rangle$, due to the application of either a short cavity pulse at the start of the protocol, or continuous drive. Here, we provide a detailed discussion on the techniques used to obtain the theoretical predictions in Figs. 5 and 6 (see solid curves in both figures). For more details on using this general framework to extract master equation parameters and experimental validation based on continuous wave measurement, see Sec. IV D and Sec. 1 E, respectively.

Our goal is to solve the effective quantum master equation under a cavity linear drive, which can be written in rotating frame for both the qubit and the cavity, with qubit rotates at its bare frequency ω_q, cavity and cavity linear drive rotates at the reference frequency ω_r as

$$\partial_t \hat{\rho} = -i \left[\Delta_c \hat{a}^\dagger \hat{a} + \frac{\lambda}{2} \hat{\sigma}_z \hat{a}^\dagger \hat{a} + \hat{H}_{\mathrm{dr}}, \hat{\rho} \right] + \mathcal{L}_{\mathrm{diss}}^{(\mathrm{eff})} \hat{\rho}, \tag{S46}$$

where the linear drive on the cavity is described by the Hamiltonian $\hat{H}_{\mathrm{dr}} = \epsilon(t)(\hat{a}^\dagger e^{-i\Delta_{\mathrm{d}} t} + h.c.)$, the dissipative term $\mathcal{L}_{\mathrm{diss}}^{(\mathrm{eff})}$ is defined in Eq. (S31) (see also Eq. (2) in the main text), and Δ_c (Δ_d) denotes detuning between cavity mode a frequency (drive frequency) and reference frequency, $\Delta_c = \omega_c - \omega_r$ ($\Delta_d = \omega_{\mathrm{dr}} - \omega_r$). Note that for the qubit Ramsey experiments with a pulsed cavity drive, the cavity drive is set to be resonant with $\Delta_d = 0$. While the following discussion is applicable to pulsed and continuous drives alike, to simplify notations, we abbreviate the drive amplitude $\epsilon(t)$ as ϵ when it does not cause confusion. We first go into the cavity rotating frame with respect to drive detuning Δ_{d}, so that we have

$$\begin{aligned}\partial_t \hat{\rho} = & -i \left[(\Delta_c - \Delta_d) \hat{a}^\dagger \hat{a} + \frac{\lambda}{2} \hat{\sigma}_z \hat{a}^\dagger \hat{a} + \epsilon(\hat{a}^\dagger + \hat{a}), \hat{\rho} \right] \\ & + \mathcal{L}_{\mathrm{diss}}^{(\mathrm{eff})} \hat{\rho},\end{aligned} \tag{S47}$$

The qubit coherence function $\langle \hat{\sigma}_-(t) \rangle$ can thus be computed by solving the following equation of motion ($\langle \uparrow | \hat{\rho} | \downarrow \rangle \equiv \hat{\rho}_{\uparrow\downarrow}$)

$$\begin{aligned}\partial_t \hat{\rho}_{\uparrow\downarrow} = & -i \left[(\Delta_c - \Delta_d) \hat{a}^\dagger \hat{a} + \epsilon(\hat{a}^\dagger + \hat{a}), \hat{\rho}_{\uparrow\downarrow} \right] \\ & - i \frac{\lambda}{2} \left\{ \hat{a}^\dagger \hat{a}, \hat{\rho}_{\uparrow\downarrow} \right\} + \mathcal{L}_{\mathrm{diss}}^{(\mathrm{eff})} \hat{\rho}_{\uparrow\downarrow}.\end{aligned} \tag{S48}$$

Before we proceed, it is worth noting that, in principle, one could try to simulate Eq. (S48) with a brute-force approach, by directly numerically evolving the quantum master equation in the joint basis between qubit states $|\uparrow\rangle$, $|\downarrow\rangle$ and cavity photon Fock states. In this case, the simulation cost would depend on the transient cavity photon number, and grows as the cavity pump power is increased. Here, we take a approach whose computation cost does not scale with photon number, making use of the fact that Eq. (S48) describes a quadratic superoperator on the coherence operator $\hat{\rho}_{\uparrow\downarrow}$, so that Gaussian operators stay Gaussian. As a result, the dynamics in Eq. (S48) could be solved making use of standard phase-space-based methods [54]. However, noting that the dissipative dynamics in Eq. (S31) do not involve any heating dissipator, this procedure can be further simplified, by taking the ansatz

$$\hat{\rho}_{\uparrow\downarrow}(t) = |\bar{a}_\uparrow(t)\rangle \langle \bar{a}_\downarrow(t)| . \tag{S49}$$

Substituting Eq. (S49) into Eq. (S48), one can show that when the conditional amplitudes satisfy the following linear equations of motion, Eq. (S48) is also satisfied

$$i\partial_t \bar{a}_{\sigma_z} = \mathcal{E}_{\sigma_z} \bar{a}_{\sigma_z} + \epsilon, \quad (\sigma_z = \uparrow, \downarrow), \tag{S50}$$

where the effective energies of the conditional cavity amplitudes can again be calculated using Eq. (S32), but with the drive detuning $\Delta_c - \Delta_d$ replacing Δ_c. Note that the first term in the right hand side of Eq. (S50) can be intuitively viewed as taking the expectation value of the Langevin equation (S26), ignoring the noise terms. In this case, we can also calculate the qubit coherence function via the equation below

$$\begin{aligned}\frac{d\langle \hat{\sigma}_-(t) \rangle}{dt} & = \Lambda \bar{a}_\uparrow \bar{a}_\downarrow^* \langle \hat{\sigma}_-(t) \rangle \\ & = \left[-i\lambda + \Gamma(e^{i\theta} - \cosh \eta) \right] \bar{a}_\uparrow \bar{a}_\downarrow^* \langle \hat{\sigma}_-(t) \rangle,\end{aligned} \tag{S51}$$

where the coefficient Λ is given in Eq. (S33). Note that we have reduced the problem of simulating the equation of motion (S48) of an operator acting on the cavity Hilbert space, down to integrating the set of linear differential equations in Eq. (S50), which greatly improves the efficiency of the numerical calculations.

Interestingly, under coherent cavity drives, the instantaneous Stark shift and dephasing rates of the qubit, corresponding to the real and imaginary components of $\Lambda \bar{a}_\uparrow \bar{a}_\downarrow^*$ in Eq. (S51), maintain their relative ratio under magnetic field

inversion $B \to -B$. This arises from the Onsager-type symmetry relations for the phase of Λ:

$$\frac{\Lambda(B)}{|\Lambda(B)|} = \frac{\Lambda(-B)}{|\Lambda(-B)|}. \tag{S52}$$

One can further show the mode amplitudes $\bar{a}_\uparrow, \bar{a}_\downarrow^*$ are symmetric due to their dependence on the field-symmetric self-energy terms $\mathcal{E}_\uparrow, \mathcal{E}_\downarrow^*$ (c.f. Eq. (S50)). As a result, the time-cumulative quantity $\frac{\ln \zeta}{\phi}$, as discussed in the main text in Figs. 6 and 7, is field-symmetric under coherent drives.

To prove Eq. (S52), we rewrite Eq. (S24) relating Λ to response properties of the intermediary modes in terms of the effective cavity self energies $\mathcal{E}_{\sigma_z}$, making use of Eq. (S21), so that we have

$$\begin{aligned}\Lambda &= \frac{\chi_0 \left|G_{21}^{(0)}\right|^2}{\left\{G_{11}^{(0)} - \frac{\chi_0}{2}\det G_{\{1,2\}}^{(0)}\right\}\left\{G_{11}^{(0)*} + \frac{\chi_0}{2}\det G_{\{1,2\}}^{(0)*}\right\}} \\ &= \frac{\chi_0 \mathcal{E}_\uparrow \mathcal{E}_\downarrow^*}{1 - \left(\frac{\chi_0}{2} G_{22}^{(0)}\right)^2}\left|G_{21}^{(0)}\right|^2. \end{aligned} \tag{S53}$$

From Eqs. (S39) and (S44) we see that all terms on the right-hand side of Eq. (S53) are symmetric with respect to the magnetic field except for the real-valued prefactor $|G_{21}^{(0)}|^2$. Therefore, the phase of both sides of Eq. (S53) must be symmetric with respect to $B \to -B$ as well.

E. Simulation of the effective master equation with a continuous or pulsed cavity drive

From Eqs. (S49) and (S51), the system dynamics under continuous drive can be straightforwardly solved. In this case, the drive amplitude ϵ in Eq. (S50) is given by a constant, so that in the stationary state regime, the qubit-state-conditional cavity amplitudes can be solved by setting the right hand sides of Eq. (S50) to zero. Substituting these steady-state amplitudes into Eq. (S51), we can analytically derive the qubit Stark shift (Δ_q) and dephasing rate (Γ_q) as

$$i\Delta_q - \Gamma_q = \frac{-i\lambda + \Gamma(e^{i\theta} - \cosh\eta)}{\mathcal{E}_\uparrow \mathcal{E}_\downarrow^*}\left|\epsilon^2\right|. \tag{S54}$$

Invoking this equation, and noting that the conditional cavity frequencies now explicitly depend on the drive detuning Δ_d, we can compute the theory curves in Fig. 5.

To produce the theoretical prediction (solid curves) for the case with pulsed cavity drive in Fig. 6, we implement a square cavity pulse of width 18 ns in Eq. (S50) before the beginning of the qubit measurement sequence, and then evolve Eq. (S50) without driving (i.e. setting $\epsilon(t) = 0$) for the duration of the measurement. We then use the numerically solved conditional cavity amplitudes and Eq. (S51) to compute the time evolution of qubit coherence function. When comparing the theory predictions with the experimental values, we introduce an additional shift in the zero of the temporal axis, to account for the relatively longer qubit $\pi/2$ pulse during the Ramsey sequence.

F. Simulation of the effective master equation with an initial Fock state, or quasi-single photon dynamics

Here we discuss the numerical methods used to obtain the theoretical prediction in Fig. (7), i.e. simulating Eq. (2) either from a single-photon Fock state or from a qubit excited state with an additional four-wave mixing (FWM) pulse. For the first case, the qubit coherence function $\langle\hat{\sigma}_-(t)\rangle$ can similarly be computed by solving the following equation of motion

$$\partial_t \hat{\rho}_{\uparrow\downarrow} = -i\left[-\Delta_c \hat{a}^\dagger \hat{a}, \hat{\rho}_{\uparrow\downarrow}\right] - i\frac{\lambda}{2}\left\{\hat{a}^\dagger \hat{a}, \hat{\rho}_{\uparrow\downarrow}\right\} + \mathcal{L}_{\text{diss}}^{(\text{eff})}\hat{\rho}_{\uparrow\downarrow}. \tag{S55}$$

With the cavity starting from a single-photon Fock state as the initial state, the qubit coherence function in the long-time steady state limit can be analytically derived as

$$\frac{\langle\hat{\sigma}_-(\infty)\rangle}{\langle\hat{\sigma}_-(0)\rangle} = \frac{\kappa + \Gamma e^{i\theta}}{i\lambda + \kappa + \Gamma\cosh\eta}, \tag{S56}$$

which lets us compute the yellow squares in Fig. 7.

For the FWM case, the qubit is initialized into a state with nonzero population in the higher-level $|f\rangle$ state, and the total system then evolves in the presence of the FWM drive that enables the $|f0\rangle \leftrightarrow |g1\rangle$ transition. In the rotating frame with respect to the FWM drive frequency, the total system master equation can thus be written as

$$\begin{aligned}\partial_t \hat{\rho} = &-i\left[\hat{H}_{\text{eff}} + \Omega(\hat{a}^\dagger |g\rangle\langle f| + \text{H.c.}), \hat{\rho}\right] + \mathcal{L}_{\text{diss}}^{(\text{eff})}\hat{\rho} \\ &+ \gamma_f \mathcal{D}\left[|e\rangle\langle f|\right]\hat{\rho} + \gamma_e \mathcal{D}\left[|g\rangle\langle e|\right]\hat{\rho}. \end{aligned} \tag{S57}$$

where $\hat{H}_{\text{eff}}$ and $\mathcal{L}_{\text{diss}}^{(\text{eff})}$ are again the effective Hamiltonian and dissipator after adiabatic elimination of the other waveguide and circulator modes (see Eqs. (S30) and (S31)), and Ω denotes the FWM drive Rabi frequency. To accurately describe time evolution in this case, we also need to include the T_1 decay processes of the excited $|f\rangle$ and $|e\rangle$ states, with corresponding decay rates given by γ_f and γ_e respectively.

In the experiments, the cavity mode a starts in the vacuum state, whereas the qubit starts in a mixture between $|f\rangle$ and $|g\rangle$ states due to initialization imperfections. In this case, time evolution according to Eq. (S57) only involves a small finite number of system levels, so that its numerical integration is straightforward. Making use of the numerical solution to Eq. (S57), we obtain the theory predictions for an imperfect initial $|f\rangle$ state (purple diamonds) in Fig. 7.

2. EXPERIMENTAL SETUP AND TECHNIQUES

A. Device and measurement setup

The device is mounted under a mezzanine plate which is thermalized to the MXC plate of a Bluefors LD250 dilution refrigerator, as shown in Fig. S1, and is positioned at the center of a superconducting solenoid magnet which operates at 4 K. The qubit port and cavity port of the device are connected

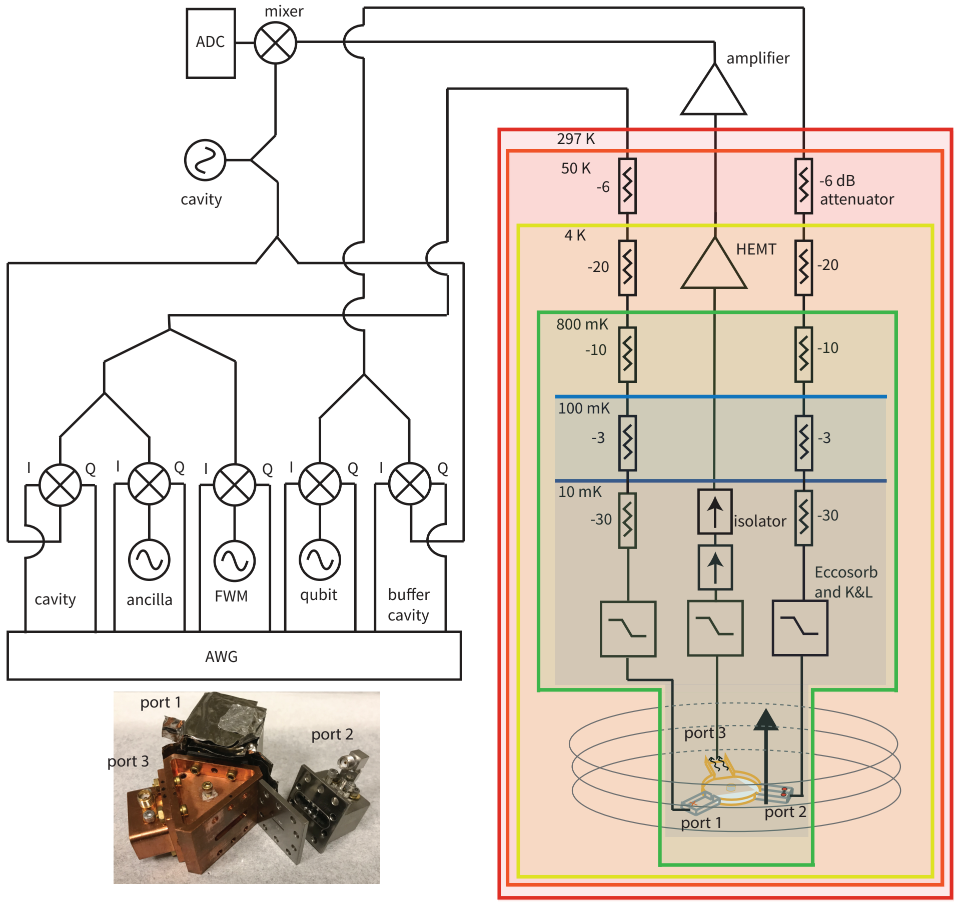

FIG. S1. **Fridge wiring schematic.** Our experiment is performed in a Bluefors LD250 dilution refrigerator. The device is mounted under a mezzanine plate which is thermalized to the MXC plate whose base temprature is around 10 mK. The device is positioned at the center of a superconducting solenoid magnet which operates at 4 K. The qubit port and cavity port of the device are connected to input cables (with attenuators as marked) and the open waveguide port is connected to an output amplifier line. The right bottom corner is the picture of the actual device, two niobium segments are hooked to a copper intermediary region via niobium plates with apertures to illicit an evanescent coupling. A 21.5 mm $\times$ 5.5 mm sapphire chip hosting a qubit is inserted into the niobium segment slot, with extra indium added at the end of the sapphire slot to ensure the chip is held in place. Two extra blank chips are inserted to maintain the cavity frequency and the symmetry of the electromagnetic field to ensure enough coupling to the intermediary mode. Two tuning screws at the top of the niobium cavity allow fine tuning of the cavity frequency. Both niobium cavities are shielded with extra layer of mu-metal to provide further magnetic protection for the qubits.

to input cables (with attenuators as marked) with eccosorb filters and a 12 GHz K&L low pass filter to filter high frequency niose and protect the qubit. The open waveguide port is connected to an output amplifier line, so that we can get the cavity transmission measurement results through the output port. The cavity port is used to drive both the cavity and its ancilla which is dispersively coupled to the cavity. The cavity state information is reflected through the read out ancilla state. The qubit port receives a drive for both the qubit and its buffer cavity, with the buffer cavity's transmission signal collected from the same output port. The qubit is dispersively coupled to the buffer cavity, allowing it to act as a proxy for information about the cavity state. The FWM drive, which is for the quasi-single photon measurements, is sent from a separate generator to the cavity port as well.

Both cavities are made out of niobium which is superconducting at low temperature and remains superconducting under the magnetic field applied for this experiment. These cavities are shielded with mu-metal sheets to further protect the qubits from the applied magnetic field, as shown in the inset picture of Fig. S1. The mu-metal sheets are mounted to the cavity for proper thermalization.

Qubits are integrated with the setup to perform this experiment, as shown in the inset picture in Fig. S1, by using a 21.5 mm $\times$ 5.5 mm sapphire chip. This chip hosts a qubit and is inserted into the niobium segment slot, with extra indium added at the end of the sapphire slot to ensure the chip is mechanically held in place and properly thermalized. Note that there are three sapphire chips in the niobium segment, and the qubit is located at the rightmost chip. The rest of the chips are there for maintaining the symmetry of the electromagnetic field of the cavity mode inside and tuning the cavity mode frequency. To further tune the frequency, two tunning screws at the top of the niobium cavity are used to fine tune the cavity

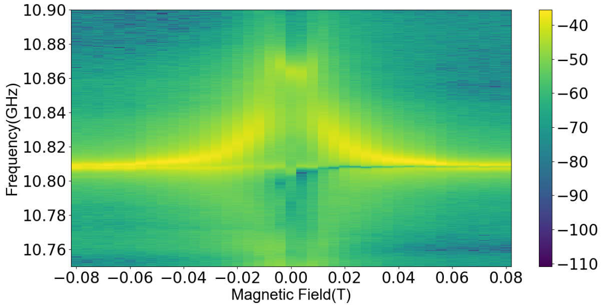

FIG. S2. **Device transmission measurements under various external fields.** Transmission coefficient S_{31}, from the buffer cavity port to the output port, measured with a vector network analyzer under various external magnetic fields. The dominant peak at each field is the buffer cavity mode which is broadband at low field due to the strong coupling to the output port. The dip at around 10.81GHz corresponds to the cavity mode.

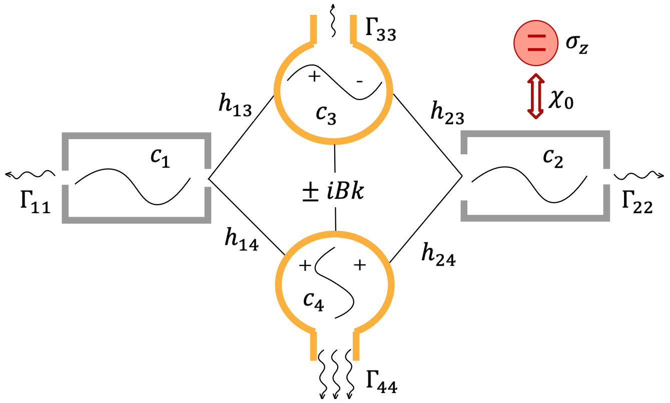

FIG. S3. **An ideal model for the intermediary modes.** This simple 4-mode model (see Eq. (S58)) includes the cavity (c_1), the buffer cavity (c_2, which is strongly coupled to the qubit) and two quasi-degenerate modes in the Y-junction region (c_3 and c_4). The degeneracy between c_3 and c_4 is lifted by the external field which is modelled through the coupling term $\pm iBk$, where B is the magnetic field and k is a constant.

frequency, thus obtaining a cavity frequency of 10.808 GHz and the qubit's buffer cavity frequency 10.809 GHz. As mentioned in the main text, the niobium segment housing the qubit we are interested in is connected to the center region through a plate with 8 mm × 1 mm aperture, which makes this niobium segment a very broadband cavity. It has a larger than 5 MHz linewidth at all tested fields with a linewidth above 20 MHz at lower fields. The operating cavity uses a 2.5 mm radius circular aperture, making it a relatively low linewidth.

The transmission properties of the device are obtained through a Vector Network Analyzer (VNA), with the VNA ports connected to the buffer cavity port and the output port. Sweeping the fridge magnet, we obtain the spectrum in Fig. S2.

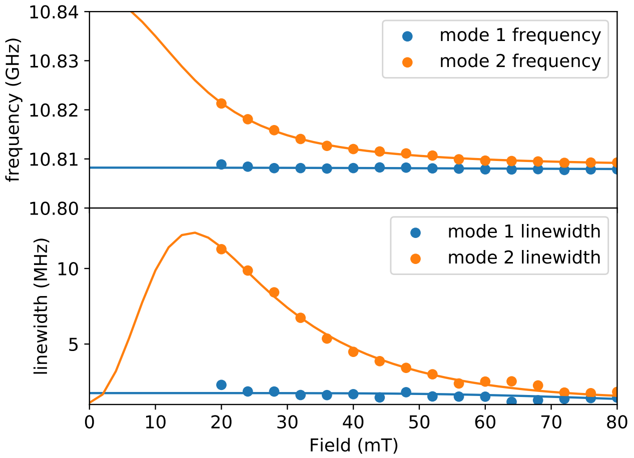

FIG. S4. **Fitting parameters of the eigenmodes used to reconstruct the 4-mode model (Eq.** (S58)**).** Plotted data points are eigenmode frequencies and linewidths as extracted by fitting the measured linear response function to a set of Lorentzians, whereas the curves represent theory predictions using Eq. (S58). The effective Hamiltonian parameters used to produce the curves are: $h_{11}/2\pi =$ 10.8085 GHz, $h_{22}/2\pi =$ 10.8076 GHz, $h_{33}/2\pi =$ 10.746 GHz, $h_{44}/2\pi =$ 11.173 GHz, $\Gamma_{11}/2\pi =$ 1.5 MHz, $\Gamma_{22}/2\pi =$ 0.3 MHz, $\Gamma_{33}/2\pi =$ 0.0 MHz, $\Gamma_{44}/2\pi =$ 476.6 MHz, $h_{13}/2\pi =$ 13.5 MHz, $h_{14}/2\pi =$ 5.6 MHz, $h_{23}/2\pi = -58.9$ MHz, $h_{24}/2\pi =$ 24.3 MHz, $k = 13.7$.

B. A simple model for the intermediary modes

To provide some additional insight to the physical structure of the bath modes, here we present a simple toy model based on our understanding of the ideal cavity-magnon polariton mode pair with participation from the Kittel mode (uniform magnetization precession) only, assuming a linear magnetic susceptibility for YIG in the range of magnetic fields of interest (at least a factor of 2 below magnetization saturation) [26]. Realistic mode structures are impacted by magnetic crystalline anisotropy of YIG (which is insignificant at room temperature but we found to be significant at low temperature), degeneracy-breaking geometric imperfections of the cavity and the YIG cylinder, nonlinearity of the YIG magnetization curve, and should also include contributions from higher-order magnon modes and dielectric resonance modes of the YIG cylinder.

We focus on the qubit and four main linear modes in our system, that is, the cavity c_1, the buffer cavity c_2, two quasi-degenerate modes in the Y-junction region c_3, c_4, as shown in Fig. S3. The treatment of the linear components is similar to that in our previous work [26]. More concretely, the intrinsic dynamics of the linear modes can be described via the

following 4×4 non-Hermitian matrix:

$$\frac{H_{\text{eff}}}{\hbar} = \begin{pmatrix} h_{11} - i\frac{\Gamma_{11}}{2} & 0 & h_{13} & h_{14} \\ 0 & h_{22} - i\frac{\Gamma_{22}}{2} & h_{23} & h_{24} \\ h_{13} & h_{23} & h_{33} - i\frac{\Gamma_{33}}{2} & -ikB \\ h_{14} & h_{24} & ikB & h_{44} - i\frac{\Gamma_{44}}{2} \end{pmatrix}. \tag{S58}$$

The parameters in Eq. (S58) can be determined, by fitting the transmission spectrum to the measured data. Specifically, the real and imaginary parts of the complex eigenvalues of Eq. (S58) correspond to the observed Lorentzian frequencies and half linewidths, respectively.

This model proved insufficient in capturing all the relevant features of the transmission data over the entire range of magnetic fields we measured. As we will discuss, the high field data is more suitable for such fitting procedure and constraining the parameters in Eq. (S58), and we fit the extracted Lorentzian parameters (frequencies and linewidths) of the two prominent eigenmodes in Fig. S2 with the predictions of the 4×4 Hamiltonian model at higher fields as shown in Fig. S4. From this fitting we can determine all the free parameters in the model Eq. (S58). Importantly, the main decay channel of the device is large enough, with an intermediary mode decay rate $\Gamma_{44} = 476.6$ MHz, for us to perform adiabatic elimination and derive an effective qubit-cavity master equation model at higher fields ($B > 20$ mT). Note that the 4×4 microscopic model in Eq. (S58) with this set of extracted parameters does not capture well the transmission property of our device at zero field, where the theoretical model would predict a narrower linewidth for mode c_2, clearly contradicting the broadband resonances as observed in the transmission measurement in Fig. S2.

We now introduce the qubit, which can shift the buffer cavity frequency by the direct dispersive shift, as denoted by χ_0. With a concrete microscopic model of the system in Eq. (S58), we can compute the qubit evolution with an equator state as the initial state and the cavity c_1 starting in a coherent state. We plot the predictions of the qubit Stark shift χ_{cq} and dephasing per photon in the dashed lines in Fig. S5. While these show a relatively similar trend as our experimental observations, they do not match quantitatively. This is a clear sign of the simple 4-mode model failing to capture the full system dynamics; in contrast, the master equation model discussed in the main text and Sec. 1 does not require knowledge about the microscopic bath modes and provides a quantitatively accurate description of the system dynamics.

Starting from the 4-mode model and employing adiabatic elimination, we derive the whole set of the master equation parameters as plotted in Fig. S6, which show a reasonable agreement for θ, η. Based on this set of derived parameters, we can further predict the χ_{cq}, χ_{qc} and dephasing per photon via the solid lines in Fig. S5, which show a good agreement with the 4-mode model predictions and also exhibiting relatively similar trends as the experimental observations. As expected, the effective 1-mode model established in this manner does not describe the experiments quantitatively well, as Eq. (S58) only provides an ideal model for the actual mediating bath of the cavity-qubit system.

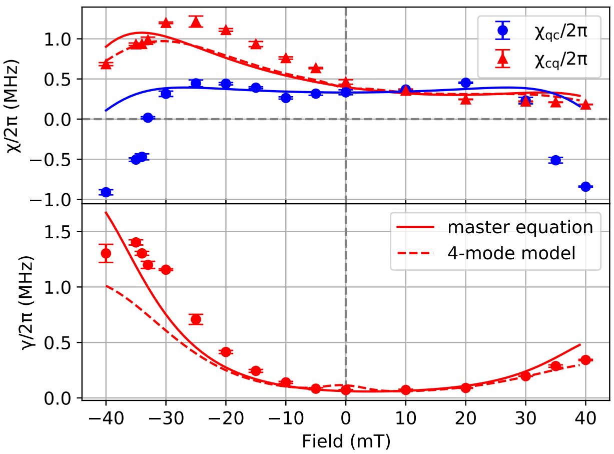

FIG. S5. **4-mode model and master equation predictions of qubit χs and dephasing.** The top panel is plotting both χ_{qc} and χ_{qc} and the bottom panel is plotting hte dephasing rate γ. The points are from experimental data and the dashed line is the prediction from the 4-mode model. The master equation prediction, plotted as a solid line, uses parameters derived from the 4 mode model. All prediction plots show a reasonable trend agreement to the data but does not provide great accuracy in all field ranges.

C. Ramsey measurement protocol and analysis

Using the measurement protocol as shown in Fig. 2(A), the full qubit Ramsey results at 30 mT and -30 mT are obtained and shown in Fig. S7. The cuts of $t = 200$ ns is shown in Fig. 2(B), which can be fit to a simple sinusoid to accurately extract the amplitude and phase of the qubit coherence function $\langle \sigma_-(t) \rangle$. By comparing $\langle \sigma_-(t) \rangle$ to the reference qubit state $\langle \sigma_-^0(t) \rangle$ measured without cavity photons (right panels) at the same t, we can extract the photon-induced phase shift ϕ and decoherence factor ζ at time t from: $\langle \sigma_- \rangle / \langle \sigma_-^0 \rangle = \zeta e^{i\phi}$. Where the measured phase shift is dominated by the term $\Gamma \sin \theta$ of the master equation as per Eq. (16). The decoherence rate is dominated by the term $\Gamma(\cosh \eta - \cos \theta)$, see Eq. (17). We repeat this analysis for all of the delay time cuts, and obtain the time domain values of qubit coherence (ζ), accumulated phase shift (ϕ) and the ratio beetween qubit decoherence and phase shift ($\ln(\zeta)/\phi$) of qubit Ramsey measurement results for transient dynamics plots in Fig. 6.

Cavity Ramsey measurements, using the measurement protocol in Fig. 2(c), provide oscillatory data that is fit with an exponentially decayed sinusoid. The fitted oscillation frequency thus determines the cavity frequency. Note that we do not obtain the cavity decay rate based on the fit exponential decay rate, as the y-axis of the oscillation is directly related to the ancilla state. While this oscillation monotonically depends on the photon number, it is not linearly proportional. The cavity decay rate measurement follows the pulse diagram in Fig. 4(A). The cavity decay rates, i.e. cavity T_1 information,

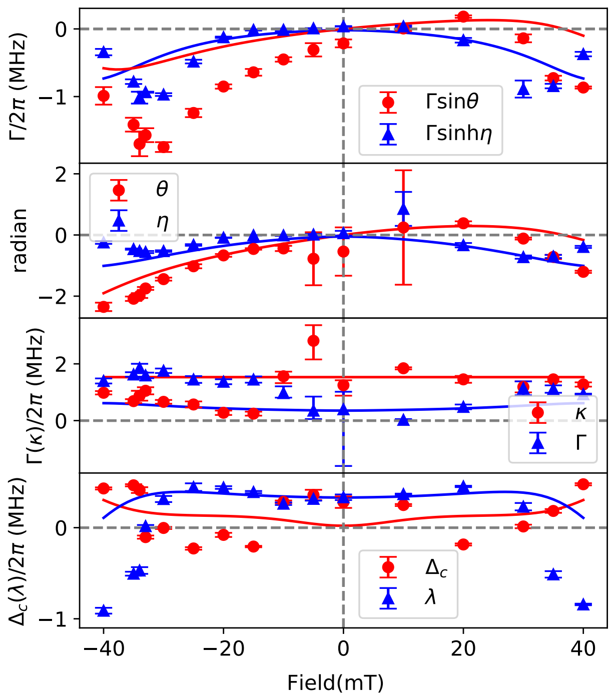

FIG. S6. **4-mode model prediction of master equation parameters.** The solid lines represent the master equation parameters derived from the 4-mode model with adiabatic elimination, whereas the experimentally derived parameters are plotted as dots. The θ, η, display a pretty good match, thus showing our understanding of the intermediary modes with the 4-mode model gives reasonable, but not perfect, predictive power to the master equation parameters.

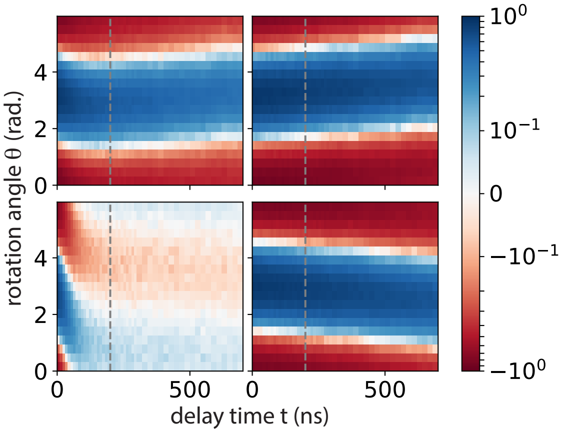

FIG. S7. **Qubit Ramsey measurement results at** ± 30 **mT.** We plot the raw result of qubit Ramsey measurements, showing the qubit state σ_z under different delay times as the x-axis (t in Fig. 2(A)). The y axis is the second qubit pulse's rotation angle (θ in Fig. 2(A)). The upper panels depict the data taken at 30 mT while the lower panels are taken at -30 mT. The left panels show evolution under cavity photons population and right panels show just the natural qubit evolution. The plot in Fig. 2(B) is the depicted dashed linecut at $t = 200$ ns.

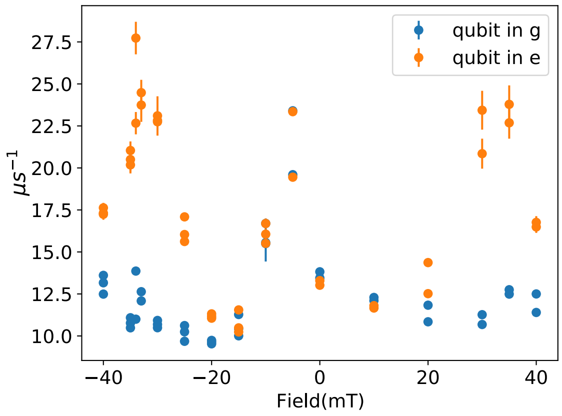

FIG. S8. **Cavity decay rate across magnetic field.** The plotted cavity decay rates are based on the same measurement protocol and analysis as shown in Fig. 4 across varying magnetic fields.

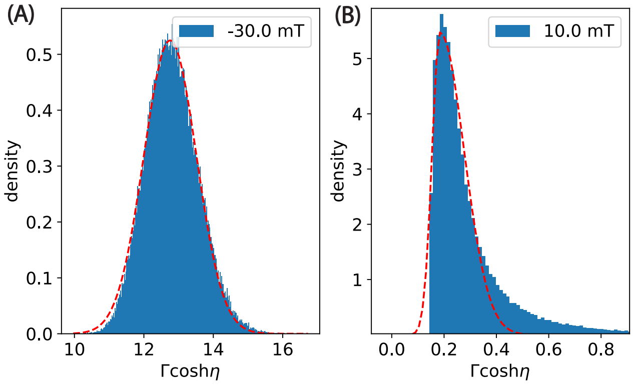

FIG. S9. **Gaussian fits to obtain uncertainty of master equation parameters.** Master equation parameter distributions utilizing a Monte Carlo simulation for the uncertainty extraction for (A) $\Gamma \cosh \eta$ at -30 mT yield a fairly good Gaussian distribution; the Gaussian fit curve (red dashed line) yields $\sigma = 0.76$ as its uncertainty. The extraction for (B) $\Gamma \cosh \eta$ at 10 mT, a noticeably asymmetric distribution, involves the combination of two half Gaussian fit curves for each side (red dashed line), yielding $\sigma = 0.03$ from the left half curve as a lower bound uncertainty and $\sigma = 0.09$ from the right half curve as a higher bound uncertainty.

are obtained and plotted in Fig. S8. These values can then be used, along with the other measured values, to determine the master equation parameters as laid out in Sec. IV D and shown in Fig. 3.

D. Master equation parameter uncertainty estimation

The analytical expressions used to compute the master equation parameters, Eqs. (12)-(17), lead to nonlinear propagation of uncertainties from the experimental observables to the model parameters that may incur singularities. Thus, we perform bootstrapping with Monte Carlo simulations to extract uncertainties for each model parameter. Assuming each

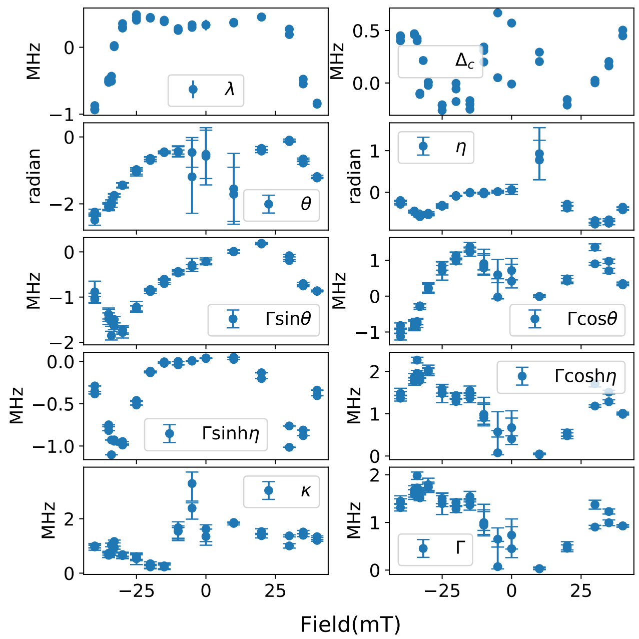

FIG. S10. **Master equation parameters.** The master equation parameter values and uncertainties from measurements are plotted. Each set of measurement data is used in a Monte Carlo simulation to determine the associated uncertainties.

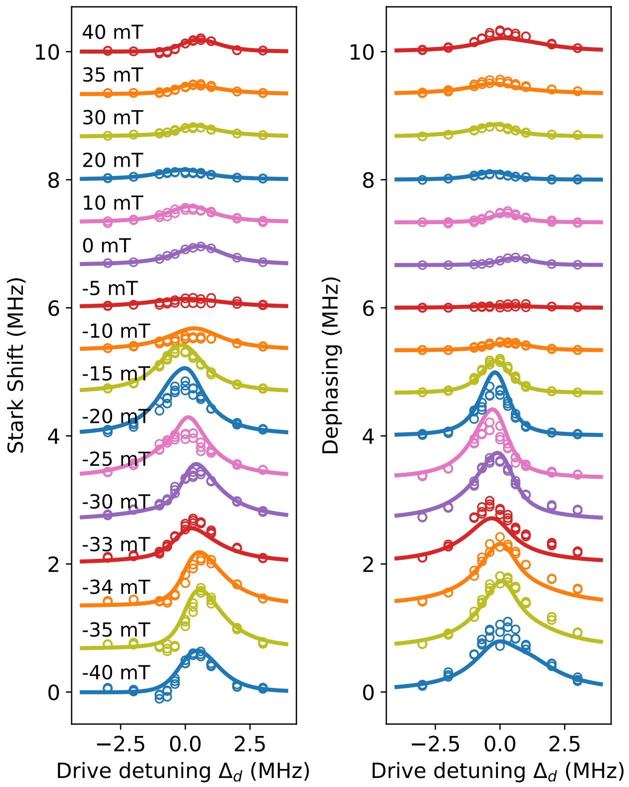

FIG. S11. **Complete data set for parameter-free verification of the master equation model with continuous cavity drive.** Theory prediction (solid lines) and experimental result (dots) of qubit Ramsey measurement under a continuous cavity drive are plotted, as shown in main text Fig. 5. The left panel depicts the qubit Stark shift and right panel the dephasing rate, both across magnetic field. Note that the theory predictions contains no free parameters and show good agreement to the experimental data.

measurement output satisfies a Gaussian distribution, with the standard deviation σ of the Gaussian distribution set by the corresponding measurement uncertainty, we perform 10^5 simulation runs with randomly generated input to accumulate the output value statistics of the master equation parameters based on Eq. (12)-(17). For cases shown in Fig. S9(A), where the master equation parameter generated fits well to a Gaussian distribution, the standard deviation σ of the Gaussian curve fit is used as the uncertainty for this parameter. For cases where the distribution shows asymmetry like Fig. S9(B), where a simple Gaussian distribution cannot provide an accurate fit, we did separate half Gaussian fits for the left and right to obtain the uncertainty of the upper and lower bounds of the parameter. The fitting result of the Gaussian center would be the value obtained for the master equation parameter value for each set of the measurements, as plotted in Fig. S10.

Due to repeated measurements at the same magnetic field being performed, the result in Fig. S10 has multiple data points. We can then average these points to get a final value and uncertainty at a specific field. There is an intrinsic data point uncertainty and an uncertainty associated with the distribution of the data points, we combine these two contributions in the reported uncertainty assuming they are uncorrelated.

E. Validation of the master equation predictions with continuous wave (CW) cavity drive

In Fig. S11, we show the complete data set for qubit Ramsey measurements under a continuous cavity drive, as shown in main text Fig. 5. We include the theory prediction and experimental results for all of the fields, to highlight their agreement and thus provide more support for the parameter-free verification of the master equation model.

3. EXPERIMENTAL RESULTS ON DEVICE B

In addition to the results shown in the main text, we performed a set of measurements on a slightly different device, which we will refer to as Device B. This device has similar cavity parameters and different qubit parameters, the main difference being the much smaller dispersive shift χ_0 between the qubit we are interested in and the broadband cavity that is housing it. The whole set of qubit and cavity parameters for both devices are shown in Table S1.

We observe the nonreciprocal frequency shift from Device B, as shown in Fig. S12. We then repeat the procedure of master equation parameter extraction on this setup, and plot the results in Fig. S13, to provide further verification for the master equation (2). There is a discrepancy from the prediction that κ is symmetric based on theory as it appears to have

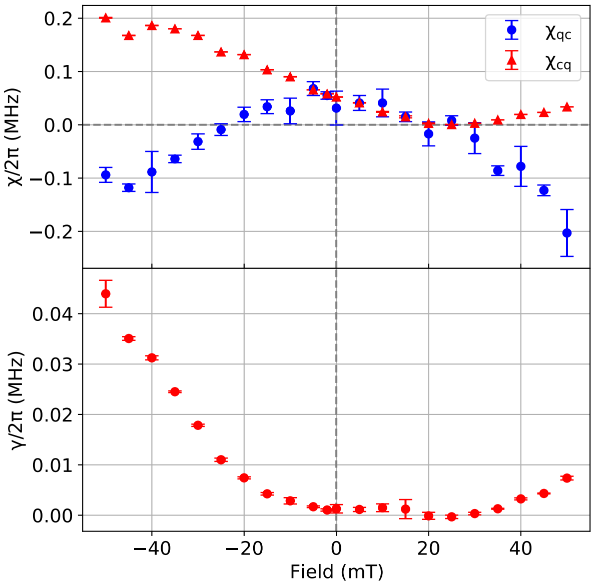

FIG. S12. **Nonreciprocal frequency shift and dephasing data for Device B.** The upper panel plots measured χ_{cq} and χ_{qc} under different external magnetic fields, showing variable nonreciprocity between the cavity and the qubit. The $\chi_{\rm qc}$ is near-symmetric across the external field and the $\chi_{\rm cq}$ is asymmetric. The lower panel shows the qubit dephasing rate under different external magnetic fields.

qubit	frequency	8.305 GHz	$\chi_0/2\pi$ 0.7 MHz
	anharmonicity	492 MHz	
	T_1	12.7 μs	
	T_2	5.5 μs	
	T_2 Echo	10.1 μs	
buffer cavity	frequency	10.812 GHz	
	linewidth	> 5 MHz	
cavity	frequency	10.814 GHz	$\chi_a/2\pi$ 1.1 MHz
	linewidth	1.5 - 2.0 MHz	
ancilla	frequency	9.173GHz	
	anharmonicity	272 MHz	
	T_1	0.6 μs	
	T_2	0.8 μs	
	T_2 Echo	0.8 μs	

TABLE S1. Experimental parameters for the Device B measurements.

a degree of asymmetry in our measurement results. We believe this could be explained by the higher frequency modes that exist in the cavity and can be excited by the cavity drive as well. These modes are coupled to the complex intermediary modes that dephase and Stark shift the qubit. We believe those effects induced by the high frequency modes are symmetric with respect to different external fields. At negative fields, the dephasing and Stark shift effects from our aimed cavity is large, making this extra contribution negligible. However, at positive fields, such contribution from the higher frequency modes could become dominant. This would result in an overestimation of $\Gamma\cosh\eta$ that depends on qubit dephasing measurements, and an over- or underestimation of

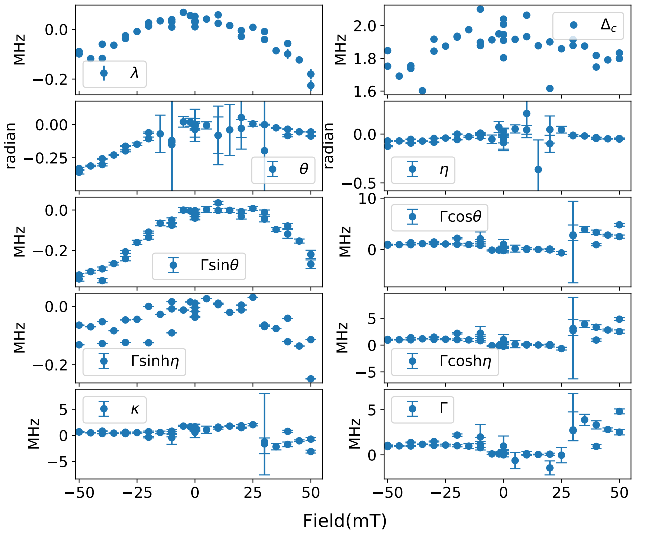

FIG. S13. **Master equation parameters extracted for Device B.** The extracted master equation parameter values and uncertainties for setup 2 are plotted utilizing the same Monte Carlo simulation method as the first set of experiments.

the absolute value of the nonreciprocal frequency shift $\Gamma\sin\theta$, which depends on the difference of $\chi_{\rm qc}$ and $\chi_{\rm cq}$. The net effect amounts to a mischaracterization of Γ at negative external fields, which in turn leads to a discrepancy in the extracted value of κ (see Eq. (15)). We believe both the deviation of measured κ (in both setups) from the symmetric field dependence as predicted by theory, as well as the unphysical values of κ extracted for Device B, could be attributed from the aforementioned effect.

With the fully extracted model, we theoretically compute the qubit Stark shift and dephasing rate under continuous cavity drive and compare them to experimental results. Interestingly, the master equation still produces relatively reasonable predictions, even though κ can now take unphysical values. Moreover, as Fig. S14 shows, the theoretical lineshapes of qubit frequency shift and dephasing exhibit a nontrivial asymmetry with respect to drive detunings, which matches the asymmetry observed in experimental results. This is reasonable as what controls the cavity decay is the sum, $\kappa+\Gamma e^{\eta\sigma_z}$, which depends on qubit state but in principle does not require precise knowledge of κ on its own.

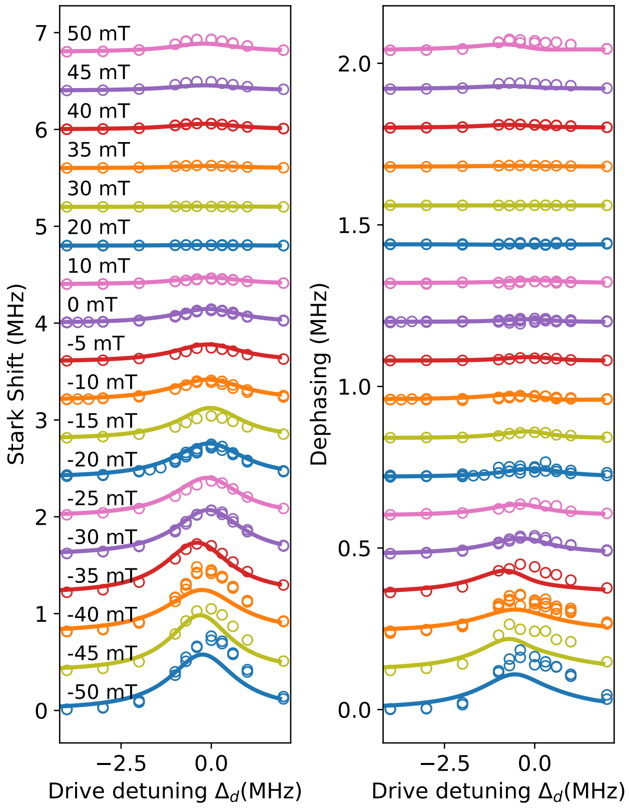

FIG. S14. **Complete data set of Device B for parameter-free verification of the master equation model with continuous cavity drive.** The theory predictions (solid lines) and experimental results (dots) of the Device B qubit Ramsey measurement under a continuous cavity drive are plotted(see main text Sec. II D for discussion on experimental protocol and analysis used). Note that the theory predictions contain no free parameters and show good agreement with the experimental data for field traces at lower fields, i.e. within 30 mT.
